# Neurocomputational mechanisms underlying the subjective value of information

**DOI:** 10.1038/s42003-021-02850-3

**Published:** 2021-12-13

**Authors:** Ariel X.-A. Goh, Daniel Bennett, Stefan Bode, Trevor T.-J. Chong

**Affiliations:** 1 Turner Institute for Brain and Mental Health, Monash University, Melbourne, VIC 3800 Australia; 2 School of Psychological Sciences, Monash University, Melbourne, VIC 3800 Australia; 3 Department of Psychiatry, Monash University, Melbourne, VIC 3800 Australia; 4 Princeton Neuroscience Institute, Princeton University, Princeton, NJ 08540 USA; 5 Melbourne School of Psychological Sciences, University of Melbourne, Melbourne, VIC 3010 Australia; 6 Department of Neurology, Alfred Health, Melbourne, VIC 3004 Australia; 7 Department of Clinical Neurosciences, St Vincent’s Hospital, Melbourne, VIC 3065 Australia

**Keywords:** Human behaviour, Decision, Motivation, Motivation

## Abstract

Humans have a striking desire to actively seek new information, even when it is devoid of any instrumental utility. However, the mechanisms that drive individuals’ subjective preference for information remain unclear. Here, we used fMRI to examine the processing of subjective information value, by having participants decide how much effort they were willing to trade-off for non-instrumental information. We showed that choices were best described by a model that accounted for: (1) the variability in individuals’ estimates of uncertainty, (2) their desire to reduce that uncertainty, and (3) their subjective preference for positively valenced information. Model-based analyses revealed the anterior cingulate as a key node that encodes the subjective value of information across multiple stages of decision-making – including when information was prospectively valued, and when the outcome was definitively delivered. These findings emphasise the multidimensionality of information value, and reveal the neurocomputational mechanisms underlying the variability in individuals’ desire to physically pursue informative outcomes.

## Abstract (web summary)

In this study, authors investigate the neurocomputational mechanisms that drive an individual’s subjective preference for information. Using fMRI and model-based analysis, they demonstrate that information value is multidimensional, accounting for the individual variability in the pursuit of informative outcomes.

## Introduction

Seminal studies in information processing have shown that humans and other animals consistently pursue information even if it cannot be utilised to improve future outcomes^1–8^. This is in striking contrast with traditional theories of reward maximisation, which propose that information is only valuable when it has instrumental utility (i.e., is useful for obtaining other rewards or primary reinforcers) that outweighs its costs^9^. Several frameworks suggest that the intrinsic value of information may be quantified along multiple dimensions, including the capacity of that information to (1) reduce uncertainty; and (2) generate desirable beliefs (i.e., its expected valence)^10,11^. Although there is considerable data on how the brain estimates these features, most current models assume that such estimates are computed in a similar manner across participants. Importantly, however, individuals vary widely both in their subjective estimates of uncertainty, and their desire for positively valenced information^1,5^. These elements together modulate the importance, or subjective value, that individuals place on the information, and their desire to physically pursue it^12^. However, the neural mechanisms underlying this individual variability remain unclear.

At the core of many current theories of information-seeking is the axiom that the desire to seek information reflects a desire to reduce uncertainty^1,5,10,13^ (although see^14,15^). Such frameworks define the value of information as the amount of uncertainty (or entropy) that it has the capacity to reduce^1,5,12,16^. Typically, uncertainty is defined according to the Shannon entropy of beliefs—a function from information theory that stipulates a fixed relationship between entropy and objective outcome probabilities^17^. However, the assumption that individuals perceive uncertainty in a fixed manner is at odds with behavioural observations that individuals vary widely in their preference to reduce it^1,5,18^. Despite the fact that perceived uncertainty lies at the core of many extant models of information value, an outstanding question is how individuals vary in their subjective estimates of uncertainty, and their tolerance of it.

Debate has also centred on how information value is driven by its expected valence. In general, humans exhibit a bias towards obtaining information that they expect will be positive versus negative. For example, individuals may prefer to remain ignorant about results from a medical test when the outcome may be potentially negative versus positive^19,20^, or about the value of their stock portfolio in a downward-trending market^6^. However, models of uncertainty reduction predict that any information that reduces uncertainty should have value, regardless of its expected valence^3,4,21^. This is consistent with findings that rats and humans prefer early information about an upcoming electric shock even if it is unavoidable^7^, and that there is an overall preference for information regardless of whether it conveys a positive or negative outcome^1,11^. The issue of how the valence of information modulates information value is therefore yet to be definitively addressed.

In determining how individuals subjectively value information, a useful approach is to examine the sacrifices that they are willing to incur in exchange for it. To date, the majority of studies have asked participants to decide on the amount of money they would be willing to sacrifice in return for information^1,5,6^ (although some have used temporal delays^22^). A limitation of monetary and temporal costs is that, in either case, a small initial increment of each cost substantially discounts the willingness of individuals to choose the information at hand. Similarly, individual differences in price elasticity (i.e., marginal value of money) also affect preference for information, thereby impairing the ability to measure individual differences in the value of information itself^23^. These factors could potentially reduce the sensitivity of models to capture individual differences in information valuation^1^. An alternative cost that has been shown to discount rewards more gradually and incrementally is physical effort^24–29^, which may offer a sensitive approach to examining cost-benefit trade-offs in acquiring information.

Neuroimaging studies have shown that information-seeking is associated with activation in brain regions often implicated in reward-based decision-making^3,4,30^. These include the ventromedial prefrontal cortex (vmPFC)^12,22^, orbitofrontal cortex (OFC)^2,6,31^; anterior cingulate cortex (ACC)^32,33^; and ventral striatum (VS)^6,12,32^. Importantly, few studies have focussed on the processes underlying the subjective estimates of information value^12^, particularly across its multiple dimensions (such as how individuals estimate and tolerate uncertainty, and how they value positively valenced information). Consequently, it remains unclear which areas encode the value of information across multiple stages of decision-making: from the prospective valuation of information to be received, to the actual delivery of the information itself. This is an important question, given that areas that are fundamental to information-seeking behaviour may be predicted to hold representations of subjective information value in a similar manner across multiple stages of the decision process.

In this model-based neuroimaging study, we applied a novel information-seeking paradigm to determine the neurocomputational mechanisms underlying the subjective value of information across its component dimensions. Participants in our study had to decide how much physical effort they were willing to invest for advanced information about an unchangeable lottery outcome. Importantly, information in our task was non-instrumental, which allowed us to separate the intrinsic value of information from any potential utility to alter future outcomes. We systematically varied the initial probability of winning the lottery on each trial, which allowed us to examine the effect of both uncertainty and expected valence on the desire to seek information. By estimating the subjective value of information across multiple stages of decision-making—from when it was prospectively evaluated to when it was definitively delivered—we were able to determine the brain regions critical to information value.

To anticipate our results, our computational models revealed that the best-fitting model of information value incorporated three key parameters: the sensitivity of individuals to uncertainty; the subjective value of reducing that uncertainty; and the subjective value of positive over negatively valenced information. Critically, fMRI data revealed that a single node within the anterior cingulate cortex encoded the subjective value of information both when prospectively valued, as well as when the outcome related to that information was actually delivered.

## Results

**Fig. 1:**
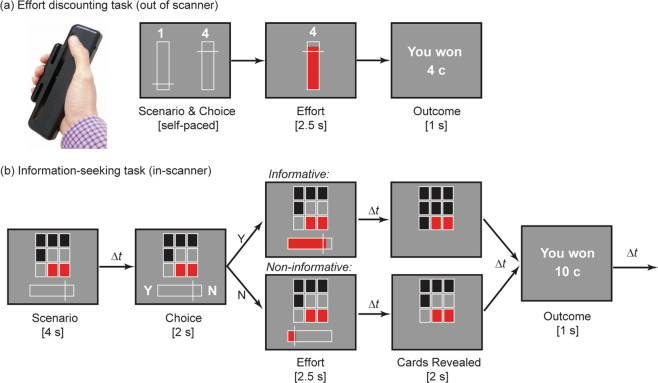
Trial structure for the effort-discounting, and information-seeking tasks. **a** In the effort-discounting task, participants chose between a fixed low-reward/low-effort baseline (left of screen), and a variable high-reward offer (right of screen) associated with an equal or higher level of effort. Effort was indicated as the height of a target line on a vertically-oriented force bar. Participants received real-time feedback of their force exertion, before the outcome of their choice was revealed. **b** In the information-seeking task, participants had to decide whether they were willing to invest effort to obtain advanced information regarding an unchangeable lottery outcome. Each lottery involved a set of nine red or black cards, and participants won the lotteries in which the majority of the cards belonged to a predesignated winning colour (here, black). In the ‘Scenario’ event, participants were presented a subset of cards, and were asked whether they were willing to invest effort to reveal the identity of the remaining cards. Higher effort levels were depicted further to the right of a horizontal force bar. They were told that their choices would not alter the final outcome of the lottery, which was predetermined. At the ‘Choice’ event, participants could either choose to reveal the remaining cards for that effort level (‘Y’), or remain ignorant about the remaining cards for minimum effort (‘N’). If participants chose to reveal the cards, they exerted their chosen level of effort (the ‘Effort’ event), and the hidden cards were revealed (the ‘Reveal’ event). If participants chose not to reveal the cards, they exerted minimal effort, and the same starting configuration of cards as in the ‘Scenario’ event was displayed. The final outcome of the lottery was presented at the end of the trial (‘Outcome’). Δt indicates jitters of 3–6 s.

The critical task was an information-seeking paradigm, in which participants had to decide how much physical effort they were willing to invest in return for non-instrumental information (Fig. 1). We operationalised physical effort as the amount of force exerted on a hand-held dynamometer, and defined six different effort levels as proportions of each individual’s maximum voluntary contraction (MVC, as defined at the beginning of the study). Twenty-six young, healthy adults performed this task while being scanned with fMRI. To confirm the efficacy of our effort manipulation, and to accurately model each individual’s sensitivity to effort costs, participants performed a standard physical effort-discounting task outside the scanner^25,27,34^. The information-seeking task in the scanner and the effort-discounting task were closely matched in their effort requirements and overall task structure, and were performed in counter-balanced order (see “Methods”).

### Behavioural results

#### Effort-discounting task

**Fig. 2:**
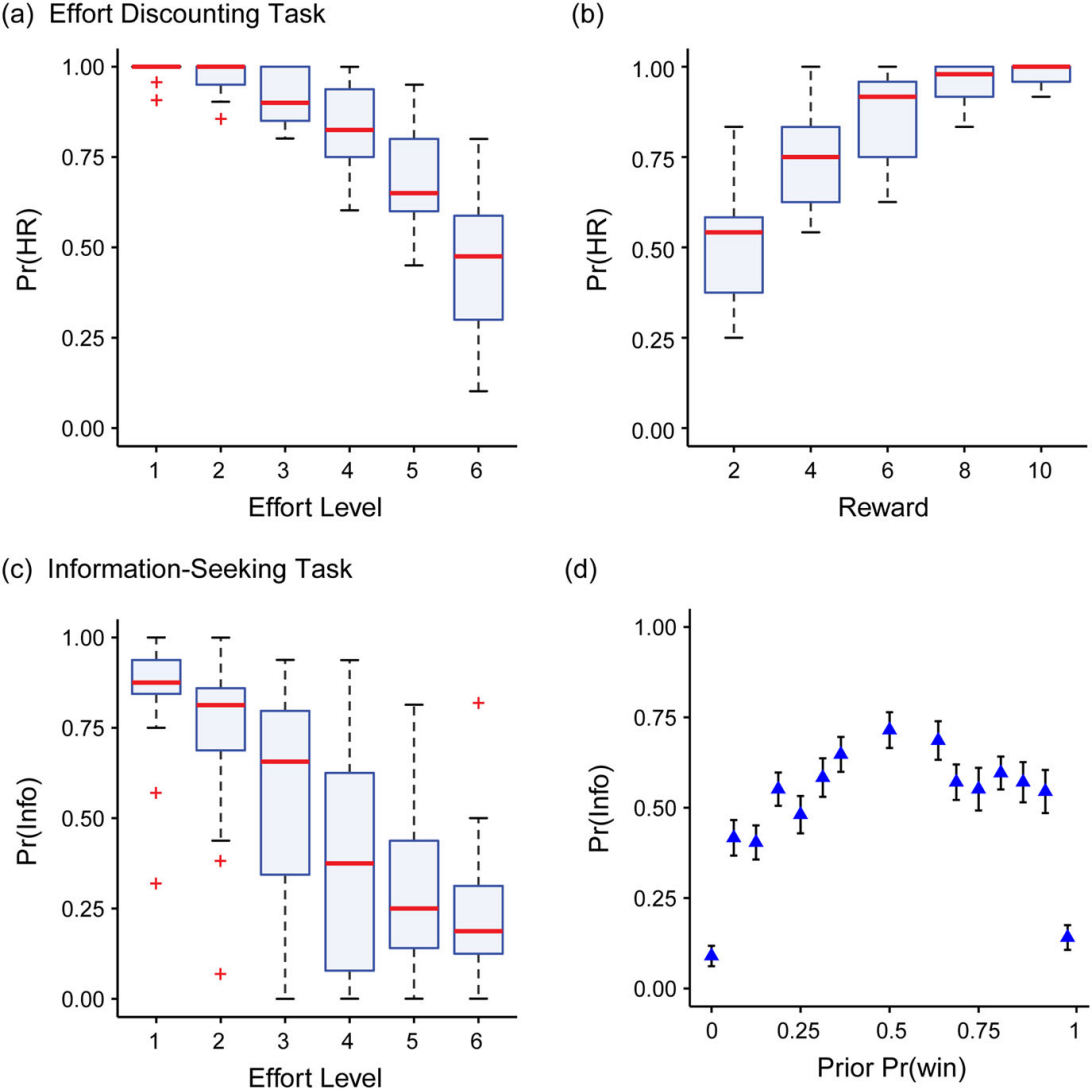
Behavioural choice data for the effort-discounting and information-seeking tasks. **a**, **b** Proportion of trials in the effort-discounting task in which participants chose the High Reward option (Pr(HR)), as a function of the (**a**) effort and (**b**) reward on offer. The red lines indicate the condition median, and the boxes extend over the interquartile range (25th to 75th percentile). The whiskers extend to the largest value no further than 1.5 times the interquartile range. Outliers (i.e., those whose data lie beyond 1.5 × the interquartile range) are indicated by red ‘+’s. (**c**, **d**) Preference for the informative option in the information-seeking task (Pr(Info)), plotted as a function of: (**c**) effort, and (**d**) the prior probability of winning, Pr(Win). Triangles depict group means, and error bars one standard error of the mean.

In the effort-discounting task, participants had to choose between a fixed, low-effort/low-reward baseline versus a more lucrative offer (Fig. 1a). The low-reward option involved exerting minimal effort (Level 1) to earn 1 cent. In contrast, the more lucrative offer required individuals to exert an equal or higher level of effort (Level 1–6) to earn 2 to 10 cents. We performed a mixed-effects logistic regression on choice data by modelling the fixed effects of: (1) the Effort and (2) Reward of the more lucrative offer, and (3) Task Order (effort discounting vs information-seeking first); and allowed random intercepts for participants. As expected, the probability of choosing the more lucrative offer was greater with decreasing Effort (*χ*^2^(1) = 169.50, *β* = −3.25, *p* < .001; Fig. 2a), and increasing Reward (*χ*^2^(1) = 94.65, *β* = 3.47, *p* < .001; Fig. 2b). The order in which participants completed the tasks did not influence choice (*χ*^2^(1) = 0.62, *β* = 0.49, *p* = .43). Overall, this confirmed the efficacy of our effort manipulation.

#### Information-seeking task

In the information-seeking task, participants were presented with a lottery on every trial, the outcome of which was determined by the majority colour amongst a set of nine black or red cards. If the majority of cards belonged to a predesignated winning colour (e.g., black), participants would win 10c; otherwise, they would win nothing (Fig. 1b). At the beginning of the trial (the ‘Scenario’ event), participants were shown a subset of cards from the full set of nine, and had the option to either: (1) gain advanced information about the ultimate outcome of the lottery (the ‘informative’ option), or forego such information, and wait until the end of the trial to discover the outcome (the ‘non-informative’ option). Choosing either option required the exertion of effort. Importantly, however, the non-informative option required only minimum effort (Level 1), whereas the informative option required effort that was equal to or greater than the non-informative option (Levels 1–6). Furthermore, we emphasised to participants that the outcome of each lottery was predetermined, and that their decisions could not influence those outcomes. Thus, any information gained by effort was entirely non-instrumental, as it only affected participants’ certainty regarding the lottery outcome.

During the ‘Scenario’ event, we systematically varied the number and proportion of revealed cards, which allowed us to manipulate both the initial level of uncertainty (maximal when Pr(win) = 0.50, and minimal when Pr (win) = 0.0 or 1.0), and the expected valence of information (positive when Pr (win) > 0.50, and negative when Pr (win) < 0.50). Participants registered their preference for the informative or non-informative option through a button press with their left hand (‘Choice’). Participants were provided with a motor cue that mapped onto the corresponding response, with mappings randomly assigned on each trial. If they chose the informative option, they were required to exert the required level of effort (‘Effort’). We then revealed the full card set (‘Reveal’), before providing them with the monetary outcome (‘Outcome’). If they chose the non-informative option, participants had to exert the minimum amount of effort. No further information was provided, however, and participants had to wait for the final ‘Outcome’ event for the lottery outcome to be revealed.

To verify that effort had a comparable effect on discounting information value as it did on reward, we conducted a mixed-effects logistic regression, with participants as a random effect, in which we modelled the fixed effects of five factors. We modelled the Prior Probability of Winning (Pr (win)), as well as its squared value (Pr (win)^2^) given that the effect of Pr (win) on choice was hypothesised a priori to be concave down (i.e., minimal when uncertainty was low; and maximal when uncertainty was high). We also examined whether participants simply estimated information content based on the raw amount of visual information on the screen (the number of cards initially revealed), which served as a rough proxy for the mathematically defined amount of information. Including this regressor allowed us to ensure that participants were actually considering the amount of information, rather than solely adopting a simpler heuristic whereby they sought information if there were fewer cards initially displayed. Finally, as in the effort-discounting task, we analysed the effects of Effort requirements, and Task Order.

This regression showed that participants chose the informative option less often with increasing Effort (*χ*^2^(1) = 56.12, *β* = −2.59, *p* < .001; Fig. 2c). In addition, they chose information more often as the probability of winning increased (Pr(win), *χ*^2^(1) = 108.94, *β* = 5.87, *p* < .001). Furthermore, they chose the informative option least often when uncertainty was minimal, and most often when uncertainty was maximal (Pr(win)^2^, *χ*^2^(1) = 116.90, *β* = −5.76, *p* < .001; Fig. 2d). Finally, they were less likely to choose the informative option as more cards were initially revealed (*χ*^2^(1) = 49.07, *β* = −0.77, *p* < .001). As for the effort-discounting task, Task Order was not significant (*χ*^2^(1) = 0.04, *β* = 0.11, *p* = .84).

Together, these logistic regressions confirmed that: (1) effort monotonically discounted both monetary reward and information value; (2) the value of an informative option was greater when the lottery outcomes were more uncertain; and (3) the value of information was greater when the expected valence of information was positive. Next, we applied a computational model of choice to determine how information itself was subjectively valued.

### Computational modelling results

Our computational modelling focused on determining whether choices were best described by models that captured individual differences in the sensitivity to uncertainty, the desire to reduce that uncertainty, and the valence of information. To obtain estimates of information-valuation parameters that were independent of estimates of effort-discounting parameters, we jointly modelled choice data from both tasks simultaneously.

#### Effort discounting

To obtain a precise estimate of subjective value, we took as our starting point a set of canonical effort-discounting functions, which considers that the subjective value of an option is a function of the reward on offer discounted by the effort involved in obtaining it. We modelled each participant’s choices in the effort-discounting task using three commonly applied functions that capture the simplest canonical patterns of effort discounting—linear, concave (parabolic), and convex (hyperbolic)—with previous work showing that parabolic functions tend to provide the best fit in dynamometer-based effort-discounting tasks^24,25,35–38^:

1 $${{{{{\rm{Linear}}}}}}:\,V{(O)}_{t}=R{(O)}_{t}-{k}_{e}\cdot E{(O)}_{t}$$

2 $${{{{{\rm{Parabolic}}}}}}:\,V{(O)}_{t}=R{(O)}_{t}-{k}_{e}\cdot E{(O)}_{t}^{2}$$

3 $${{{{{\rm{Hyperbolic}}}}}}:\,V{(O)}_{t}=\frac{R{(O)}_{t}}{1+{k}_{e}\cdot E{(O)}_{t}}$$

In these equations, the subjective value (*V*) of an option, *O*, on a given trial, *t*, is a function of its reward (*R*), and associated effort (*E*, the proportion of MVC required). The effort itself is scaled by a subject-specific effort-discounting parameter, *k*_*e*_, with higher values indicating a greater aversion to effort.

#### Information seeking

**Fig. 3:**
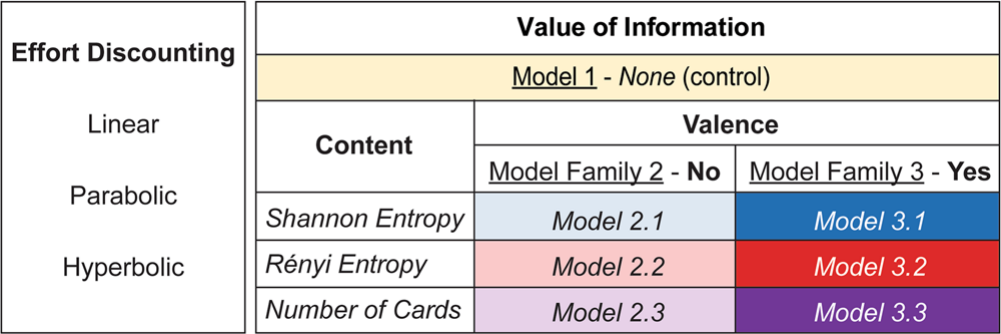
We compared 21 different models of information value. For each of three effort discounting functions (linear, parabolic, hyperbolic), we compared seven different models. Model Family 1 (yellow) was a control model, which ascribed no value to information. Model Family 2 assumed that the value of information was driven only by its content (lighter colours). Model Family 3 assumed that value of information was driven by both its content and its valence (darker colours). Each of Model Families 2 and 3 tested competing hypotheses on how the content of information is computed. Uncertainty was computed in Models 2.1/3.1 as a function of Shannon entropy (blue), and in Models 2.2/3.2 as a function of Rényi entropy (red). Models 2.3/3.3 tested the hypothesis that participants used the initial number of cards as a heuristic for information content (purple).

In the information-seeking task, we built on these models of effort discounting by incorporating the added value of information into the functions for subjective value. We took the *k*_*e*_ values defined from the effort-discounting task to determine the sensitivity of individuals to effort in the Information-Seeking task. For each of the three effort-discounting functions, we tested three main model families, which together comprised seven separate models (Fig. 3).

#### Model Family 1

This family comprised a single *control model*, which assumed that non-instrumental information has no value (as predicted by traditional reward maximisation theories). Such theories state that the only reward available is the expected monetary value of that option, *R* (i.e., 10 ¢ / 2 = 5 ¢ for all trials). Thus, the control models for the information-seeking task were identical to those for the effort-discounting tasks above, with *R = 5*.

#### Model Family 2

A second model family assumed that *the value of information is driven by its content*. Recall that the content of information is traditionally quantified based on its capacity to reduce uncertainty, which is in turn operationalised in terms of the entropy of beliefs^17,39^. Thus, this family postulates that the content of information (*I*) has some intrinsic value that varies across individuals, and that this variability can be captured by a subject-specific parameter that reflects the preference of individuals to reduce uncertainty (*k*_*i*_, −∞ < *k*_*i*_ < ∞), with positive values indicating a greater preference for information, and negative values a lower preference. The term *k*_*i*_ ∙ *I* therefore represents the subjective value that an individual places on reducing uncertainty within the environment. The value of a given option on a given trial can then be considered the effect of the expected monetary reward on offer, added to *k*_*i*_ ∙ *I*.

4 $$V{(O)}_{t}={{{{{\rm{Effort}}}}}}\,{{{{{\rm{discounting}}}}}}\,{{{{{\rm{function}}}}}}+{k}_{i}\cdot I{(O)}_{t}$$

This family comprised three specific models, each of which tested competing hypotheses on how information is subjectively valued:

Model 2.1. Shannon entropy model—Information theory formally defines the information content of an option, *I*(*O*), as the reduction in uncertainty (quantified as the entropy of beliefs, *H*), after viewing the stimulus, relative to before the stimulus was revealed:

5 $$I(O)=H{(O)}_{{{{{{\rm{prior}}}}}}}-H{(O)}_{{{{{{\rm{post}}}}}}}$$

This family of models defined entropy according to the Shannon entropy function^17^—the typical function used to quantify information value—which assumes that the relationship between outcome probabilities and uncertainty is constant across individuals:^1,5,12,31^

6 $$H(.)=-{\sum }_{j=1}^{2}{{\Pr }}({x}_{j})\cdot \,\log ({{\Pr }}({x}_{j}))$$

where {*x*_1_, *x*_2_} represents the set of discrete outcomes (*x*_1_ = win; *x*_2_ = loss).

**Fig. 4:**
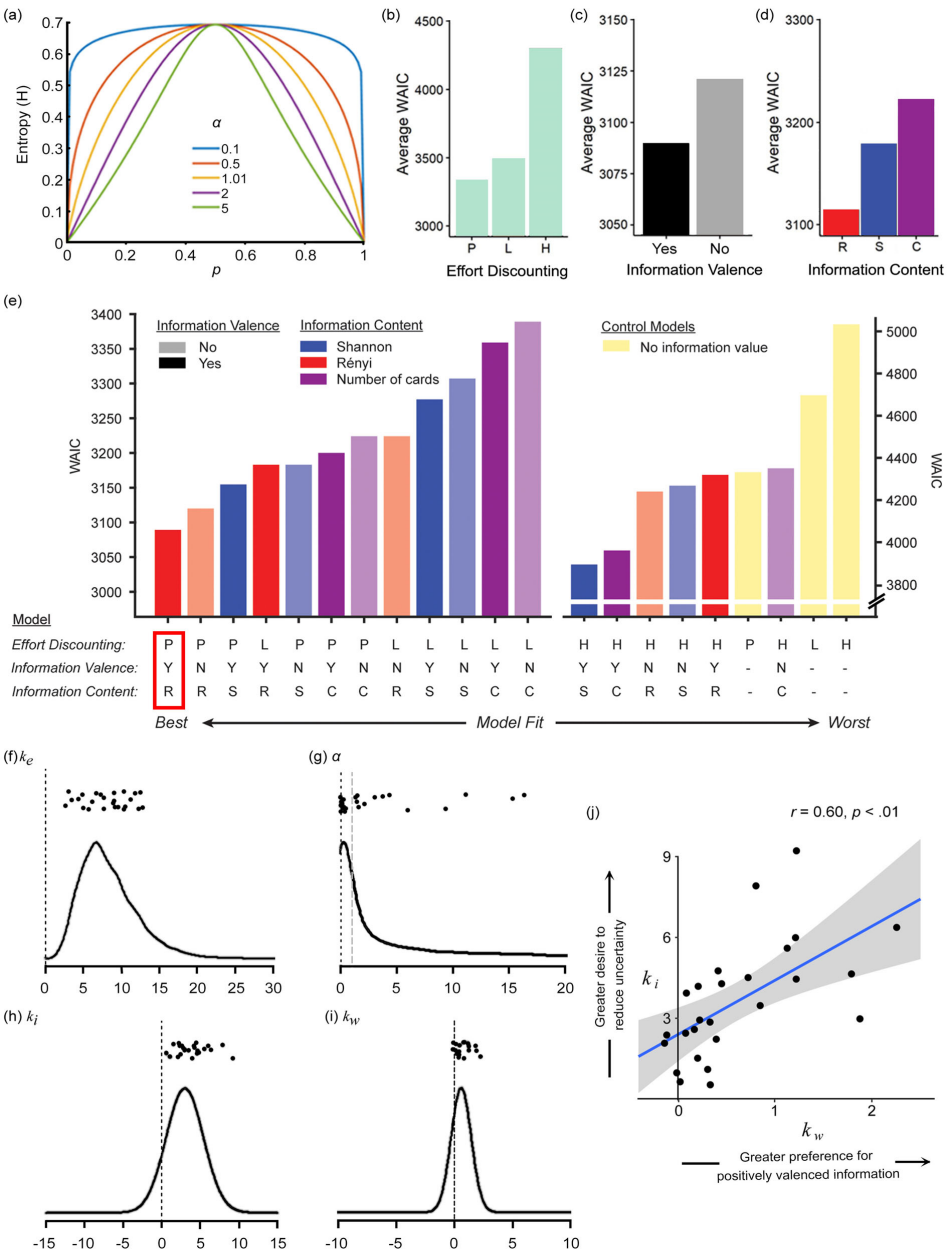
Computational modelling results. **a** Illustration of the generalised Rényi entropy function, and its capacity to flexibly estimate an individual’s sensitivity to uncertainty. Entropy varies with the probabilty, p, of a binary outcome—a relationship that is modulated by different values of the Rényi weighting parameter *α*. As *α* approaches 1, this function approximates the Shannon entropy. **b**–**d** Stepwise model selection revealed that: (**b**) Effort discounting was best fit by a parabolic function (P = parabolic; L = linear; H = hyperbolic); (**d**) The expected valence of information contributed significantly to information value; and (**d**) Information content was best quantified with the Rényi entropy function (R = Rényi, in red; S = Shannon, in blue; C = Number of cards, in purple). WAIC is presented on a deviance scale, such that lower values indicate better relative model fit. **e** Model comparisons over the entire model space confirmed that Model 3.2 was the best-fitting model. The colour key is identical to (**c**)–(**d**) and Fig. 3. Control models (assuming that information has no value) are depicted in yellow. Models incorporating an effect of information valence are depicted in darker shades, and those without in a lighter shade. Models are ordered by goodness-of-fit (note the change in scale for the poorer fitting models depicted on the right side of the panel). **f**–**i** Individual parameter estimates and inferred group-level parameter distributions from the best-fitting computational model for: (**f**) *k*_*e*_ (effort-discounting), (**g**) *α* (Rényi weighting parameter), (**h**) *k*_*i*_ (value of information content), and (**i**) k_w_ (value of information valence). Each plot visualises the distribution that is implied by using the medians of the posterior distributions of the group-level mean and standard deviation as hyperparameters. Parameter estimates for individual participants are presented above each distribut_i_on. **j** The k_i_ and k_w_ parameters were significantly correlated across participants.

Model 2.2. Rényi entropy model—A limitation of Shannon entropy is that it is unable to account for potential variability in individuals’ sensitivity to uncertain outcomes. For example, a model quantifying information in terms of reduction of Shannon entropy is constrained to treating, for all individuals, a prior win probability of Pr(win) = 0.3 as being twice as uncertain as a prior win probability of Pr(win) = 0.09. However, it is not necessarily true that all participants appraise probabilities in exactly this way. In contrast, Rényi entropy^39^ is a more generalised function, which includes a weighting parameter (α) that denotes the degree to which an individual is sensitive to uncertainty (Fig. 4a):

7 $$H(.)=\frac{1}{1-\alpha }\,\log \left({\sum }_{j=1}^{2}{{\Pr }}{({x}_{j})}^{\alpha }\right),\,{{{{{\rm{where}}}}}}\,\alpha \ge 0\,{{{{{\rm{and}}}}}}\,\alpha \, \ne \,1$$

As *α* approaches 0, all possible events are weighted more equally, regardless of their probabilities, implying a lower sensitivity to uncertainty. Conversely, as *α* approaches infinity, entropy is increasingly determined by the events of greatest uncertainty, indicating a greater sensitivity to uncertainty. Note that, as *α* approaches 1, the function approximates the Shannon entropy function. Rényi entropy therefore represents a more flexible function than Shannon entropy, and is therefore capable of capturing the variability in how individuals estimate the uncertainty of the environment.

Model 2.3. ‘Visual information’ model—Finally, we considered the possibility that information content was not related to uncertainty at all, but that participants simply used the number of cards on the screen as a heuristic for the amount of information available. Here, the value of information for an option, *I*(*O*), is simply based on the proportion of cards that are yet to be revealed:

8 $$I(O)=\frac{{{{{{\rm{number}}}}}}\,{{{{{\rm{of}}}}}}\,{{{{{\rm{unknown}}}}}}\,{{{{{\rm{cards}}}}}}}{9}$$

#### Model Family 3

A final model family tested the hypothesis that the value of information is driven by both its content and its valence. Valence, *W*, was defined as Pr(win) − 0.5. Thus, positive values of *W* indicated a higher probability of winning, and negative values a lower probability. *W* was then scaled by a parameter *k*_*w*_ (−∞ < *k*_*w*_ < ∞), which represented participants’ individual preference for positively valenced information, such that *k*_*w*_ > 0 indicated a preference for information expected to be positive, and *k*_*w*_ < 0 a preference for information expected to be negative. In this family, we assumed that valence had an additive effect on information value:

9 $$V{(O)}_{t}={{{{{\rm{Effort}}}}}}\,{{{{{\rm{discounting}}}}}}\,{{{{{\rm{function}}}}}}+{k}_{i}\cdot I(O)_{t}+{k}_{w}\cdot W{(O)}_{t}$$

Within this family, we tested the same models of information content as in Model Family 2 (i.e., the Shannon entropy model (3.1), the Rényi entropy model (3.2), and the ‘visual information’ model (3.3)).

Together, the model space therefore comprised 21 candidate models (seven for each effort-discounting function). On every trial for every participant, we used a *softmax* function to estimate the probability of choosing the informative (*I*) over the non-informative (*NI*) option on every trial:

10 $${{\Pr }}(I)=\frac{{e}^{\beta \cdot V(I)}}{{e}^{\beta \cdot V(I)}+{e}^{\beta \cdot V(NI)}}$$

where *β* is an inverse temperature parameter that defines each individual’s choice stochasticity. We fit our models to data from the effort discounting and information-seeking tasks simultaneously, holding *k*_*e*_ and *β* constant between both tasks. All models were fit using a hierarchical Bayesian approach, and Hamiltonian Monte Carlo sampling as implemented in Stan^40^. Model comparison was performed using the Watanabe-Akaike Information Criterion (WAIC)^41^.

In keeping with previous studies, the best-fitting model (Model 3.2; Fig. 4) was one that described physical effort discounting as a parabolic function (mean *k*_*e*_ = 7.75; highest density interval, HDI [6.44 9.23])^24,35,42^. Importantly, this model defined information value as a function of *both* uncertainty reduction *and* expected information valence:

11 $$V{(O)}_{t}=R{(O)}_{t}-{k}_{e}\cdot E{(O)}_{t}^{2}+{k}_{i}\cdot I{(O)}_{t}+{k}_{w}\cdot W{(O)}_{t}$$

12 $$I(O)=H{(O)}_{{{{{{\rm{prior}}}}}}}-H{(O)}_{{{{{{\rm{post}}}}}}}$$

13 $$H(.)=\frac{1}{1-\alpha }\,\log \left ({\sum }_{j=1}^{2}{{\Pr }}{({x}_{j})}^{\alpha }\right),\,{{{{{\rm{where}}}}}}\,\alpha \ge 0\,{{{{{\rm{and}}}}}}\,\alpha \, \ne \, 1$$

Importantly, information content was best modelled in terms of the Rényi entropy function. Indeed, the top two models both incorporated Rényi entropy, and differed only in their inclusion of the valence modifier (Δ WAIC = 30.96). The best-fitting model incorporating the Shannon entropy function was, overall, the third best-fitting function (Δ WAIC = 66.27). The fact that the Rényi entropy function provided the best fit indicates significant variability in how individuals estimate uncertainty. This conclusion is further emphasised by the variance of α across the group (mean 1.22; HDI [0.22 11.74]; Fig. 4g). Furthermore, this model also captured the variability in individuals’ desire to reduce their estimated uncertainty, as indicated by the positive group mean for the *k*_*i*_ parameter (mean 3.01; HDI [2.02, 3.83]; Fig. 4h). Finally, this model demonstrated that expected valence had a significant effect on information value, as emphasised by the *k*_*w*_ parameters that were positive for all except three participants, which implies an overall preference for information about expected positive versus negative outcomes across the group (HDI [0.17, 1.03], mean 0.60; Fig. 4i).

Given that this model distinguishes the value of information content (*k*_*i*_) from its valence (*k*_*w*_), we next asked whether these two parameters are related. A correlation analysis between all model parameters revealed a positive correlation between *k*_*w*_ and *k*_*i*_ (*r* = .60, *p* < .01, Bonferroni-Holm-corrected for multiple comparisons; Fig. 4j; Supplementary Table 1). This indicated that individuals with a greater preference to reduce uncertainty (i.e., with a higher *k*_*i*_) also had a greater preference for positively valenced information (a higher *k*_*w*_). This significant correlation raised the question of whether simpler models that included only a single free parameter for information value (instead of two separate parameters for *k*_*i*_ and *k*_*w*_) could provide a better and more parsimonious fit to the data. However, a simpler control model containing only a single free parameter for information performed notably worse than the winning model that included separate parameters for *k*_*w*_ and *k*_*i*_ (see “Method”).

Finally, we performed a model recovery analysis to confirm the validity of our model comparison procedure. This showed that we were able to identify the true generative model from amongst a set of similar competitors with an accuracy in excess of 95% for each model (Supplementary Table 2). We also conducted a parameter recovery analysis from the best-fitting model to ensure that its parameters were precisely recoverable. This analysis confirmed that we were able to accurately recover each of the five parameters from the wining model (all *p*-values < .001; Supplementary Table 3). Furthermore, posterior predictive checks indicated a good fit between participants’ choices and model predictions (Supplementary Fig. 1).

### fMRI results

Our key imaging question was to determine whether there are common areas that encode the subjective value of information, both when it is prospectively valued, and when it is definitively delivered. We restricted our analyses to all voxels within four regions of interest (ROIs) that form the core of the reward valuation network—the anterior cingulate cortex (ACC), ventromedial prefrontal cortex (vmPFC), orbitofrontal cortex (OFC), and ventral striatum (VS). Whole-brain analyses were conducted as additional, more exploratory analyses.

#### A cluster within the ACC encoded the prospective value of information

**Fig. 5:**
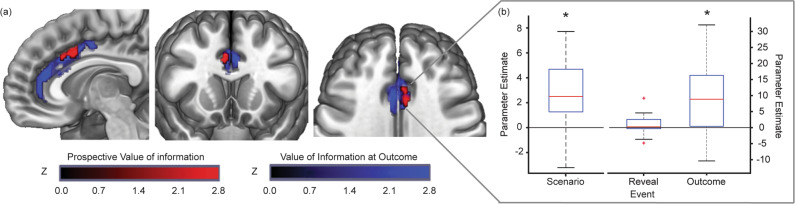
A cluster within the caudal anterior cingulate cortex encoded the subjective value of prospective information, and lottery outcomes when definitively delivered. **a** Clusters show activity that parametrically varied with the subjective value of information when prospectively evaluated (red), and when the outcome was definitively delivered (blue). The cingulate cluster that encoded information value at the delivery of the Outcome entirely encompassed the cluster that encoded the prospective valuation of information at Scenario. These results depict an analysis of all voxels within the predefined ROIs, with significant voxels indicating those that survived cluster-wise corrections for family-wise error (FWE, *p* < 0.05), with a cluster-forming threshold of *p* = 0.001 (uncorrected). No voxels encoded the value of information at the Reveal event. **b** Parameter estimates from the ACC cluster that encoded the prospective value of information at the Scenario event (i.e., the cluster shown in red in panel (**a**)). The red lines denote the median parameter estimate across the group, with the edges of each box plot indicating the 25th and 75th centiles; the whiskers extend to the most extreme data points not considered outliers, and the outliers are indicated by red ‘+’s. *, *p* <0.0005.

First, we considered areas that were engaged during the prospective valuation of information. Using the parameters from our best-fitting model, we determined the subjective value of information of the chosen option on every trial for every participant. This comprised the value related to individuals’ preference for uncertainty reduction (*k*_*i*_ ∙ *I*, with *I* defined according to Rényi entropy) added to the expected value for positively valenced information (*k*_*w*_ ∙ *W*). We took these values for every trial, and entered them as parametric modulators, time-locked to the onset of the Scenario event. This revealed a significant cluster within the caudal anterior cingulate cortex (Fig. 5, red cluster; Table 1). No activity was detected in the remaining ROIs, even at uncorrected thresholds. At a whole-brain level, other clusters that survived correction with a voxel-wise FWE rate of 0.05 included the primary motor areas, the pre-supplementary motor area, and insula/frontal operculum (Supplementary Fig. 2).

**Table 1:** Areas in which activation reflected the subjective value of information of the chosen option.

Cluster *p*_FWE_	*k*	*Z* value	*x*	*y*	*z*	Area
**Value of information—when prospectively valued**						
0.038	34	4.71	6	16	36	Caudal anterior cingulate cortex
**Value of information—at outcome**						
<0.001	509	4.44	6	38	18	Caudal anterior cingulate cortex
		3.93	4	18	30	
		3.87	−5	12	36	
0.007	72	4.42	4	44	2	Rostral anterior cingulate
		4.17	−7	42	6	

Voxels survived cluster-wise corrections for family-wise error (*p*_FWE_ < 0.05), with an uncorrected cluster-forming threshold of *p* = 0.001.

As a secondary analysis, we also considered those areas involved in the subjective valuation of the effort required for the chosen option (*k*_*e*_ ∙ *E*) (Supplementary Fig. 3). This recruited a larger, bilateral cluster encompassing both the anterior and mid-cingulate cortices. This cluster encompassed the smaller ACC region that encoded the subjective value of information.

We then decomposed the subjective value of the chosen information into its component parts, and modelled separately the value of uncertainty reduction (*k*_*i*_ ∙ *I*), and the expected value of positively valenced information (*k*_*w*_ ∙ *W*). This analysis revealed a similar cluster of activity within the ACC that parametrically varied with the value of uncertainty reduction. However, there was no significant activity that varied with the expected value of information valence, either within the ROIs, or at a whole-brain FWE-corrected level. This is most likely driven by the fact that decisions on every trial involved assessing two alternatives that differed in the amount of uncertainty that each had the capacity to reduce, but not in their expected information valence. Given that a value comparison was not required for information valence, it may be unsurprising that this value was not explicitly represented in BOLD activity. Regardless, these analyses indicate that the value of information was primarily signalled by a comparison between the capacity of both the informative and non-informative options to reduce uncertainty.

#### Medial prefrontal areas encoded the delivered value of information

Next, we considered which areas were engaged during the valuation of information when it was definitively delivered. As in the preceding analysis, we computed the value of information as a function of uncertainty reduction and expected valence (*k*_*i*_ ∙ *I* + *k*_*w*_ ∙ *W*). In our task, uncertainty could be reduced at either the Reveal stage (if the informative option was chosen), or the Outcome stage (if the non-informative option was chosen). We therefore entered *k*_*i*_ ∙ *I* + *k*_*w*_ ∙ *W* as a parametric modulator time-locked to the onset of the Reveal and Outcome event, and considered activity at each of these events separately.

Interestingly, there was no significant activity at the Reveal event within our ROIs. Across the whole brain, there was a cluster within the right fusiform gyrus that survived voxel-wise correction for family-wise error (*p*_FWE_ = 0.001; Supplementary Fig. 2). In contrast, activity at the Outcome event was present at significant thresholds across a large cluster spanning the caudal ACC. This cluster entirely encompassed the more focal ACC cluster that was engaged during the prospective valuation of information. Indeed, we found that activity within the ACC cluster engaged during the earlier Scenario event varied positively with the value of delivered information at the Outcome event (Fig. 5b). In addition, there was a separate cluster within the rostral (pregenual) ACC (Table 1, Fig. 5a, blue clusters). At the whole-brain level, activity at the Outcome event was found within areas including the right supramarginal gyrus, left inferior temporal sulcus, and right inferior frontal gyrus (*p*_FWE_ < 0.01; Supplementary Fig. 2).

Finally, a convergent finding amongst several recent studies is that there is a substantial overlap between the neural mechanisms that encode information value and reward^3–5^. Although this was not a principal question in our study, we did consider the effect of reward delivery on BOLD activity at the Reveal and Outcome stages. The main result was that the encoding of information value and reward involved regions that partially overlapped, but also those that were unique to each entity. The outcomes of this analysis are summarised in Supplementary Fig. 4 and Supplementary Table 5.

## Discussion

Individuals vary considerably in the subjective value they place on information, but the neurocomputational mechanisms underlying this valuation process remain unclear. Our key result was that the value of information depends critically on an individual’s estimates of uncertainty, their desire to reduce it, as well as their desire for positive information. Applying our computational model to fMRI data revealed that the anterior cingulate cortex was critical in prospectively estimating the future value of information, as well as the subjective value of that information when the final outcome was delivered. Importantly, a subset of voxels within the ACC encoded value at both stages of the decision-making process, suggesting a key role for this area in processing information value, and guiding information-seeking behaviour. Overall, our results emphasise the multidimensionality of information value, and reveal the key areas involved in the subjective valuation of information across multiple phases of decision-making.

Several studies have consistently demonstrated that information is intrinsically valuable, over and above any potential utility in obtaining tangible benefits^1,5,6,10,31^. The majority of studies have quantified the value of information in terms of its capacity to reduce uncertainty. Such a metric provides useful insights into how information signals can be used to update predictions about the external world. However, one important assumption of these studies is that uncertainty is estimated in a fixed manner across participants, based on an arbitrary weighting of event probabilities (i.e., according to Shannon entropy^17^). Importantly, we showed that a more generalised entropy function (the Rényi entropy^39^) was better able to capture the significant variability in individuals’ sensitivity to uncertain outcomes. In addition, participants demonstrated an overall appetite for positive information^1,5,6,10,11^, but the distribution of *k*_*i*_ values indicated substantial variability in their desire to reduce their estimated levels of uncertainty. This result has notable implications for future studies on information processing, by arguing for a more flexible approach to capture the wide range of differences in how individuals estimate uncertainty, and their tolerance of it.

Our modelling results speak to recently proposed frameworks of curiosity—the intrinsic desire of individuals to know, and to actively seek out information^43–46^. Normative frameworks of behaviour, such as the free energy principle, argue that organisms have an intrinsic tendency to minimise surprise, in order to optimise their predictions about future states of the world^47,48^. The proposition that information value is related to its capacity to reduce uncertainty fits parsimoniously into such accounts^3,4,21^. More recent frameworks, however, have highlighted the multidimensionality of information value, by arguing that information-seeking is motivated, not only by the desire to reduce uncertainty, but also by the anticipatory utility of that information^12,46^. Our findings offer support to these more recent theories, by showing that models containing a valence modifier consistently outperformed those without. This result, that the valence of information contributes positively to its value, is consistent with accounts that individuals may experience a positive emotional ‘boost’ from the anticipation of a positive outcome^49^, which may act as an additional source of internal motivation^45^ for the individual to pursue information. Indeed, some have proposed that this anticipatory utility may give information hedonic value (by inducing a positive affect), while the capacity of information to reduce uncertainty provides information with its cognitive value (by allowing an agent to update their internal models of the world)^10^.

Our data also demonstrate a strong link between separate dimensions of information value. The vast majority of participants in our task preferred positively to negatively valenced information, as evidenced by the overall positive *k*_*w*_ values across the group. This is consistent with the overall weight of evidence in the literature. Although individuals tend to exhibit a greater preference for information relating to potentially positive versus negative outcomes^6,50,51^, this is not universal, as some studies have shown that individuals demonstrate the opposite preference^52,53^. Across our sample, the *k*_*w*_ parameter was significantly positively correlated with the *k*_*i*_ parameter, indicating that individuals with a greater preference to reduce uncertainty (high *k*_*i*_) tended to be those who had a stronger preference for potentially positive information (high *k*_*w*_). This suggests that the cognitive and hedonic values of information, though distinct constructs, may nevertheless be closely related.

Our fMRI analyses built on previous neuroimaging studies which focused on where and how information prediction errors are encoded^6,31^. These previous studies have shown that areas of the reward network are also sensitive to the updating of internal predictive models to minimise uncertainty^10,54^. Although such studies have provided insight into the areas that might encode objective uncertainties in the environment, few studies have examined which areas represent the subjective value of information to the individual, particularly across its multiple dimensions^12^. We reasoned that areas that are critical to encoding the subjective value of information across its multiple dimensions should represent that value similarly when decisions are prospectively made, and when outcomes are actually delivered. Our results implicated a region within the ACC as a critical node that encodes information value at both stages, which is consistent with recent neurophysiological data demonstrating the selectivity of subpopulations of ACC neurons to the information signal^33^.

Although the activity related to information value was manifest when the Outcome was definitively delivered, it was not evident at the intermediate Reveal stage, when information was presented in the absence of the explicit lottery outcome. Although speculative, one explanation relates to the potential utility of the presented information^55,56^. At the initial Scenario event, the utility of information was obviously substantial, given that it was the key variable on which participants based their decision. At the final Outcome event, the utility of information was central to reinforcing the conditional relationship between any previously delivered information, and the result of the lottery itself. In contrast, the Reveal event was an intermediate event, at which the veracity of any delivered information had yet to be confirmed by receipt of the final outcome. This may have been particularly relevant given that all participants were naïve to our paradigm, and were unlikely to have yet formed a strong conditional relationship between the stimulus (card array) and the definitive outcome of the lottery^57–61^. Of course, this interpretation that the neural representation of information value is a function of its utility remains to be formally addressed in future experiments.

The involvement of the ACC in computing the predicted value of information, as well as the value of received outcomes, makes adaptive sense. The sensitivity of the ACC to information value at the time of the decision is consistent with previous studies showing that ACC activity reflects prospective information about a chosen option^62–64^. In addition, our result that the ACC represents the value of information on delivery of the outcome is in keeping with those of separate studies showing that the ACC indexes uncertainty^65^ and ‘surprise’ more generally when an outcome becomes known^66^. Together, representing information value within the same region at different stages of the decision process may be advantageous, as the predicted value of information within this region can then be used to compute expectations about the environment, and the value of the received outcome can then be used to update value estimates within this same region to guide future decisions^55^.

Interestingly, information value in our task was not associated with activity in our other ROIs. It is of course difficult to interpret the absence of activity, but we note that there has been some variability in the involvement of these areas in previous studies on information-seeking. For example, although there is a substantial body of work implicating the ventral striatum in processing other types of reward, its involvement in information valuation has been equivocal—whereas some studies on information value have noted ventral striatal activity^6,12^, others have not^22,31^. Similarly, although the vmPFC has been consistently implicated in reward-related processing, such studies have tended to focus on more tangible reinforcers (e.g., food, juice, or money^67–71^), and less data are available on its role in information valuation^12,22^. Importantly, information is distinct from these other reinforcers in that it tends to be associated with high outcome variance, and past studies have indeed shown a sensitivity of vmPFC neurons to outcomes with higher event probabilities^72^. Previous studies have implicated the OFC when non-instrumental information is delivered^6,31^, but not when uncertainty is initially evoked^31^. The reasons for the discrepancies between studies are not clear, and may potentially be driven by differences in task design, or, in the case of the ventral striatum, the lower signal-to-noise ratio in deeper brain structures. Nevertheless, such inconsistencies indicate potentially important points of difference in how information and other types of reinforcers are processed, and should be a focus of future work.

In summary, our findings provide insights into the neurocomputational mechanisms underlying the subjective valuation of information. A critical finding was that information value can be decomposed along several dimensions that are all subject to significant interindividual differences—including one’s sensitivity to uncertainty; desire to reduce it; and preference for information with an expected positive valence. Our neuroimaging data reveal the ACC as a key region involved in the processing of information value, both prospectively and upon receipt of the outcome associated with that information. Together, these results inform current frameworks of curiosity, which emphasise the intrinsic value of information, and the desire of individuals to pursue information for information’s sake^43–46^. Our study provides a robust method to measure individual differences in information valuation, which can potentially be used to understand deficits in curiosity that lead to sub-optimal information-seeking behaviour, both in healthy individuals and clinical populations who suffer from impairments of decision-making.

## Methods

### Participants

We recruited 30 young, healthy adults, four of whom were excluded for not understanding task instructions, leaving 26 participants in the final sample (12 male, 14 female; aged 20 to 33 (*M* = 25.19, SD = 3.25); all right-handed). As compensation, participants received a flat payment of AUD $30 plus an additional amount earned from the task (*M* = $4.83, SD = $0.32). All participants had normal or corrected-to-normal vision. All participants provided written informed consent, and protocols were approved by the Monash University Human Research Ethics Committee (ID CF16/2332-2016001170).

### Materials and procedure

Participants performed two separate tasks: a physical effort-discounting task (performed outside the scanner), and a non-instrumental information-seeking task with physical effort costs (while being scanned). Participants completed both tasks in a single session, with the order of tasks counter-balanced across participants. Stimuli were presented using the Psychophysics Toolbox implemented in MATLAB R2015b (Mathworks Inc., US). Participants held an fMRI-compatible dynamometer (SS25LA, BIOPAC Systems, USA) in their dominant (right) hand, and provided button responses with their non-dominant (left) hand.

#### Effort calibration and familiarisation

Effort in this study was operationalised as the amount of physical force applied to the hand-held dynamometer. To normalise effort requirements across tasks and across individuals, effort levels were defined as a proportion of each individual’s maximum voluntary contraction (MVC). The MVC for each participant was defined at the beginning of the study as the maximum of three successive, self-paced contractions of the dominant hand^24–27,73^. We then defined six levels of effort, ranging from 13% MVC (Level 1) to 78% MVC (Level 6), at increments of 13%. Participants were familiarised with these effort levels in a preliminary training phase, during which they had to perform a ballistic contraction to match or exceed the required effort level on each of 24 trials (4 per effort level). This training phase was conducted prior to each of the Effort-Discounting and Information-Seeking tasks.

#### Effort-discounting task

The goal of the effort-discounting task was to estimate the degree to which individuals were averse to investing effort in return for reward^27,29,34,42,74^. By assessing this behaviour separately from the information-seeking task, we were able to measure individual differences in effort discounting independently of individual differences in information valuation. On each trial, participants chose between a fixed low-reward/low-effort baseline, and a more lucrative high-reward offer that required them to invest an equal or greater amount of effort (Fig. 1a). The fixed baseline was always a reward of 1 cent for investing minimal effort (Level 1), while the variable offer was the option to win a higher reward (2, 4, 6, 8 or 10 cents) for an equal or higher level of effort (Levels 1–6). On each trial, participants viewed the Effort and Reward levels for the two options, and chose the option they thought was “more worth the effort”. Trials were self-paced, and their choice was highlighted for 0.5 s. They then had 2.5 s to exert their preferred level of effort, and were provided feedback at the end of the trial. If they successfully reached the target effort level, they were rewarded with the stake on offer; otherwise, they were rewarded 0 points. Each of the 6 Effort × 5 Reward conditions was sampled four times, for a total of 120 trials, which were divided into ten blocks to minimise the effect of fatigue.

#### Information-seeking task

To investigate preferences for non-instrumental information, we developed a novel paradigm which required participants to choose between exerting higher levels of effort to obtain predictive information about an unchangeable lottery outcome, or exerting minimum effort and foregoing such information (Fig. 1b). Each trial was a lottery comprising a set of nine black or red cards. In each lottery, participants could win 10 ¢ if the majority of cards belonged to a predesignated winning colour (e.g., black); otherwise they won 0 ¢. Importantly, the information gained by exerting higher levels of effort was entirely non-instrumental, as it only affected participants’ certainty regarding the lottery outcome, without affecting the outcome of the lottery itself, which was predetermined. Participants were informed about this feature, and confirmed that they understood that the non-instrumental nature of this information.

At the beginning of each trial, participants were shown a subset of cards from the full set of nine (the ‘*Scenario’* event)—this represented partial information about the lottery outcome. We systematically manipulated the probability of winning on each trial by varying the number and proportion of revealed cards. This allowed us to manipulate both the initial level of uncertainty, as well as the expected valence of information presented. Uncertainty was maximal when the probability of winning (Pr(win)) was 0.5, and minimal as Pr(win) approached 0 or 1. Valence was neutral when Pr(win) = 0.5; negative when Pr(win) < 0.5; and positive when Pr(win) > 0.5. We were thus able to separate out the effect of valence on equivalent levels of uncertainty. For example, the uncertainty when Pr(win) = 0.9 is identical to that when Pr(win) = 0.1, but the former has an expected valence that is positive, and the latter negative.

Pr(win) was computed as the binomial probability that the winning card colour would be in the majority^5^. This probability incorporates both the number of winning cards displayed at the Scenario event, together with all possible combinations of cards yet to be revealed:

14 $${{{{{\rm{Pr }}}}}}({{{{{\rm{win}}}}}}|n,\,{n}_{{{{{{\rm{req}}}}}}})=1-{\sum }_{k=0}^{{n}_{{{{{{\rm{req}}}}}}}-1}\left(\begin{array}{c}n\\ k\end{array}\right){0.5}^{n}$$

where *n* is the number of cards remaining to be drawn, and *n*_req_ is the number of additional winning cards required for a majority, given the number of wining cards (*n*_win_) already drawn:

15 $${n}_{{{{{\rm{req}}}}}}=\left\{\begin{array}{cc}5-{n}_{{{{{\rm{win}}}}}}, & {n}_{{{{{\rm{win}}}}}} < 5\\ 0, & {n}_{{{{{\rm{win}}}}}}\ge 5\end{array}\right.$$

This computation is the Bayes-optimal approach for computing probabilities for a binomial random variable, given the information that was available to participants^1,5^.

We chose 16 starting card configurations to sample from Pr(win) at approximately even increments (generally 0.05–0.07). This ensured that we did not oversample card configurations that led to similar values of Pr(*win*). For example, Pr(win) values between 0.64–0.69 could be the result of starting configurations comprising 3 W(inning)/2 L(osing) cards, 2 W/1 L, and 1 W/0 L. In such situations, we rationalised the number of configurations by omitting some configurations (e.g., 2 W/1 L). Note that an exception to the sampling interval of 0.05–0.07 was around Pr(win) = 0.5, in which the smallest possible increment was ± 0.14 given the nine-card structure of the task. We also ensured that, for each positively valenced (Pr(win)) configuration (e.g., 4 W/2 L), we sampled from the corresponding negatively valenced (1 − Pr(win)) configuration (i.e., 2 L/4 W). Of the 16 starting configurations, 7 were positively valenced, 7 were negatively valenced, and 2 were neutral (Supplementary Table 6). Each starting configuration was presented once at every effort level for every participant.

Participants were required to decide between one of two options—they could either remain ignorant about the concealed cards (the non-informative option), or choose to reveal all the cards to discover the lottery outcome (the informative option). Choosing either option required the exertion of some degree of effort. Importantly, however, choosing the non-informative option required individuals to exert only minimal effort (Level 1), whereas choosing the informative option required effort levels equal to or greater than the non-informative option (Levels 1–6). Note that the design of this task was closely matched to the effort-discounting task, in which participants made choices between a fixed baseline and variable offer. This included having a condition in which the informative and non-informative options were matched in effort, as a sanity-check to confirm that participants preferred informative over non-informative options when effort was not a factor. It is also worth noting that, by design, there was no ambiguity associated with the decisions that participants had to make. On each trial, both the informative and non-informative options had the same fixed probability of winning (i.e., each option was associated with the same combinations of cards). Furthermore, each option was associated with a single, known outcome—the informative option always led to the full set of cards being revealed, and the non-informative option never did.

The Scenario was displayed for 4 s followed by a random jitter of 3–6 s. Participants were then prompted to make a button press response indicating their preference (the ‘*Choice’* event). They were provided with a motor cue (‘Y’ for the informative, and ‘N’ for the non-informative option) that appeared randomly on the left or the right of the screen, and mapped onto the corresponding button press response (e.g., in Fig. 1b, informative = left button press; non-informative = right). This ensured that the Scenario event could isolate activity unique to decision-making, separate from that associated with motor preparation. Participants had two seconds in which to make their response, and their choice was then highlighted until the end of that two-second period. Immediately thereafter, participants were prompted to exert their chosen amount of effort within a 2.5 s window (the ‘*Effort’* event). If they failed to reach the required effort level in time, they automatically forfeited the lottery and received 0 cents. These two motor response screens (choice + effort) were followed by a second random jitter of 3–6 s.

Participants were then shown the set of cards they chose to view (the ‘*Reveal’* event). If participants chose the non-informative option, they were simply shown the same starting configuration of cards they had seen in the Scenario event. However, if they chose the informative option, the full set of cards was revealed for 2 s. Finally, after a further random jitter of 3–6 s, participants were provided with the monetary outcome of the lottery (‘You won: 10 ¢’ or ‘You won: 0 ¢’; the ‘*Outcome’* event). This outcome was displayed for 1 s. Note that the outcome provided complementary information to the preceding reveal—uncertainty about the lottery outcome was reduced during the Reveal event when the informative option was chosen, and during the Outcome event when the non-informative option was chosen. A further random jitter of 3–6 s was imposed between the ‘*Outcome’* event, and the ‘*Scenario’* event on the next trial.

To ensure that participants maintained task engagement, 5% of trials were catch trials, in which participants had to press any button within two seconds of a white X appearing on one of the cards. This could occur during either the Scenario or the Reveal events. If participants were successful, they would proceed to the next trial without penalty; failure to respond in time resulted in a penalty of 50 cents. Across all catch trials in all 26 participants, only one participant missed a single response (mean correct responses on catch trials across the group = 99.0%). In total, there were three runs of 32 trials (16 initial card configurations, each sampled once for each of the six effort levels). Participants completed a block of practice trials outside of the scanner before completing the main task in the scanner.

### Mixed-effects analyses

In addition to computational modelling (see below), choice behaviour on the effort-discounting and information-seeking tasks were analysed with mixed-effects logistic regression analyses using the *lme4* package in R^75^. For these analyses, we included random intercepts for all participants, as well as random slopes for all within participants predictors^76^. *p*-values for omnibus tests were computed using Wald chi-square tests with Type-III sums of squares. All continuous predictors were *z*-scored prior to analysis to allow for comparison of standardised coefficients between predictors. For all continuous regression effects, we report a chi-square statistic, a *p*-value, and a standardised regression coefficient (which can be interpreted as a change in the log-odds of the outcome variable corresponding to a one standard deviation increase in the predictor variable).

### Computational models

#### Defining information content and entropy

As discussed in the “Results”, the information content of an object, *I*(*O*), was defined as *I*(*O*) = *H*(*O*)_prior_ − *H*(*O*)_post_, where *H*(*O*)_prior_ represents the entropy of beliefs prior to the stimulus being revealed, and *H*(*O*)_post_ the entropy of beliefs after it was revealed. *H*(*O*)_prior_ was computed based on the starting card configuration presented in the Scenario display. *H*(*O*)_post_ for each of the informative and non-informative options were computed based on the capacity of each option to reduce uncertainty. The informative stimuli were associated with complete resolution of uncertainty (i.e., *H*(*O*)_post_ = 0), and their information content was therefore *I*(*O*)_info_ = *H*(*O*)_prior_. In contrast, non-informative stimuli had no capacity to reduce uncertainty (i.e., *H*(*O*)_post_ = *H*(*O*)_prior_), and their information content was therefore *I*(*O*)_non-info_ = 0. Note that all of the details that are required to compute the information gain from both the informative and non-informative stimuli were available to participants at the time of making a choice.

#### Model fitting

The 21 candidate models were fit using a hierarchical Bayesian approach. Choices from the effort-discounting and information-seeking tasks were fit simultaneously, and *k*_*e*_ and β were held constant within participants across both tasks. Model comparisons were performed using the Watanabe-Akaike Information Criterion (WAIC)^41^, a statistic for comparing models fit with hierarchical Bayesian methods. Like other information criteria (e.g., AIC, BIC, DIC), it selects models according to their goodness-of-fit (marginal likelihood, estimated as the mean log-likelihood of data across posterior samples), minus a penalty for the model’s effective complexity (estimated as the variance of the log-likelihood across posterior samples), such that more parsimonious models are favoured over more complex ones^77^.

We conducted two sets of model comparison analyses: in the first, we sought to identify the best-fitting model overall by identifying the single model with the best WAIC value. In the second, we sought to compare different model ‘families’ (i.e., those sharing features such as a common effort-discounting function or a common information-valuation function) to identify the model features that were associated with the best predictive performance across the entire model space. For the latter analysis, we compared model families by taking the mean overall WAIC across each of the models within a family.

To ensure parameters were constrained to values within an a priori plausible range, we transformed three parameters to lie within a bounded range using the cumulative normal distribution: 0 ≤ *k*_*e*_ ≤ 100; 0 ≤ α ≤ 50; and 0 ≤ *β* ≤ 20. Parameters were estimated using partial pooling, such that participant-level parameters were assumed to be drawn from group-level Gaussian prior distributions, the parameters of which were freely estimated from the data. The specific models are detailed in the “Results” section.

Given the significant correlation between *k*_*i*_ and *k*_*w*_, we performed a control analysis to verify that they truly represented distinct constructs, rather than as common manifestations of the same latent cognitive process. We formulated an additional model that was identical to our best-fitting model, with the exception that it more parsimoniously estimated *k*_*i*_ and *k*_*w*_ as a single parameter (i.e., the model was fit under the constraint that *k*_*i*_ = *k*_*w*_). Importantly, this control model was still outperformed by the original two parameter model (Δ WAIC = 268.22, SE = 37.86). This implies that *k*_*i*_ and *k*_*w*_ are best modelled as distinct processes, which were free to vary independently of each other, but happened to be closely correlated.

### Functional magnetic resonance imaging (fMRI)

#### Data acquisition

Functional MRI data were collected on a 3 Tesla Siemens Skyra MRI scanner. Stimuli were displayed on an MRI-compatible monitor positioned at the head of the scanner bore, and participants viewed the monitor through a mirror mounted on a 32-channel head coil. Functional data were acquired with a T2*-weighted gradient-echo-planar imaging (EPI) sequence using interleaved slice acquisition (T_R_ 2200 ms; T_E_ 30 ms; flip angle 90°; 38 contiguous slices with a slice thickness of 3.0 mm without an interslice gap; voxel size 3.0 mm^3^ on a base matrix of 64 × 64 pixels, oriented along the AC-PC line). In each run, we collected 455 volumes, with the first eight volumes removed to allow for steady-state tissue magnetisation. We also acquired a structural T1-weighted magnetisation-prepared rapid gradient-echo (MPRAGE) sequence for anatomical localisation (T_R_ 1,900 ms; T_E_ 2.49 ms; flip angle 9°; 192 slices with a slice thickness of 0.90 mm; voxel size 0.9 mm^3^ on a base matrix of 256 × 256 pixels), and gradient-echo field maps to correct for geometric distortions caused by inhomogeneities in the magnetic field.

#### Pre-processing

Data pre-processing was performed with FMRIPREP version stable^78^, a Nipype^79^ based tool. Each T1-weighted (T1w) volume was corrected for INU (intensity non-uniformity) using N4BiasFieldCorrection v2.1.0^80^ and skull-stripped using antsBrainExtraction.sh v2.1.0 (using the OASIS template). Brain surfaces were reconstructed using recon-all from FreeSurfer v6.0.1^81^, and the brain mask estimated previously was refined with a custom variation of the method to reconcile ANTs-derived and FreeSurfer-derived segmentations of the cortical gray-matter of Mindboggle^82^. Spatial normalisation to the ICBM 152 Nonlinear Asymmetrical template version 2009c^83^ was performed through nonlinear registration with the antsRegistration tool of ANTs v2.1.0^84^, using brain-extracted versions of both T1w volume and template. Brain tissue segmentation of cerebrospinal fluid (CSF), white-matter (WM) and gray-matter (GM) was performed on the brain-extracted T1w using fast (FSL v5.0.9^85^).

Functional data were slice-time corrected using 3dTshift from AFNI v16.2.07^86^ and motion corrected using mcflirt (FSL v5.0.9)^87^. This was followed by co-registration to the corresponding T1w using boundary-based registration^88^ with six degrees of freedom, using bbregister (FreeSurfer v6.0.1). Motion correcting transformations, BOLD-to-T1w transformation and T1w-to-template (MNI) warp were concatenated and applied in a single step using antsApplyTransforms (ANTs v2.1.0) using Lanczos interpolation.

Physiological noise regressors were extracted applying CompCor^89^. Principal components were estimated for the two CompCor variants: temporal (tCompCor) and anatomical (aCompCor). A mask to exclude signal with cortical origin was obtained by eroding the brain mask, ensuring it only contained subcortical structures. Six tCompCor components were then calculated including only the top 5% variable voxels within that subcortical mask. For aCompCor, six components were calculated within the intersection of the subcortical mask and the union of CSF and WM masks calculated in T1w space, after their projection to the native space of each functional run. Frame-wise displacement^90^ was calculated for each functional run using the implementation of Nipype.

#### Analyses

Data were analysed using SPM12 (Wellcome Department of Imaging Neuroscience, Institute of Neurology, London, United Kingdom; http://www.fil.ion.ucl.ac.uk/spm), implemented in MATLAB. Each participant’s data were modelled using fixed effects analyses. The effects of the experimental paradigm were estimated for each participant on a voxel-by-voxel basis using the principles of the general linear model (GLM). Predictor functions were formed by modelling the onsets of the events of interest with a stick (delta) function convolved with the canonical haemodynamic response function. Low-frequency noise was removed with a 128 s high-pass filter. The GLM included three regressors of interest: the Scenario event, the Reveal event, and the Outcome event, each of which was associated with a parametric modulator (see below). Other regressors which were included, but not analysed, included the motor events (i.e., the Choice and Effort events), and the onsets of the catch trials and their outcomes. The six head motion parameters derived during realignment (three translations and three rotations) were incorporated as additional nuisance regressors.

The main focus of this model-based fMRI study was to determine the neurocomputational mechanisms underlying: (1) the subjective valuation of information, and (2) the reduction of uncertainty across individual participants. To address the first goal, we computed the subjective value of information (i.e., *k*_*i*_ ∙ *I* + *k*_*w*_ ∙ *W*) for the chosen option on every trial for every participant using the parameters from our best-fitting model. As discussed in the “Results”, *I* represented the content of information, which was weighted by an individual’s preference to reduce uncertainty, *k*_*i*_; and *W* represented the valence of information, which was weighted by an individual’s preference for positively valenced information, *k*_*w*_. In addition to the value of information, we computed the subjective value of effort (i.e., *k*_*e*_ ∙ *E*) for every trial using the same model. We then entered these two subjective values as orthogonalised, parametric modulators for the Scenario event-related regressor.

To address the second goal, we computed the subjective value of information when it was finally delivered at the Reveal or Outcome screens. As for the first goal, the subjective value of information was defined through the winning model as *k*_*i*_ ∙ *I* + *k*_*w*_ ∙ *W*, which represents the amount by which uncertainty was reduced (as defined by the Rényi entropy function with a participant-specific α parameter), added to the valence of information. These subjective values were then entered as parametric modulators for the Reveal and Outcome events separately. We note that the visual displays for the Reveal and Outcome events were quite distinct, but the effects of information value as a parametric modulator was analysed for each event separately. Regression coefficients were estimated at the subject level using the standard restricted minimum-likelihood estimation implemented in SPM12. Variance inflation factors for all of our regressors were < 4, indicating that multicollinearity between regressors was not an issue in our design^91^ (Supplementary Table 4). A GLM design matrix for a representative participant is provided in Supplementary Fig. 5.

SPM contrast images from the first level were then taken to a second-level group analysis. We restricted our analyses to all voxels within regions-of-interest (ROIs) comprising the ACC, vmPFC, OFC and VS using the Harvard-Oxford Cortical and Subcortical Structural Atlas (corresponding to the ‘anterior cingulate’, ‘medial frontal’, ‘frontal orbital’ and ‘nucleus accumbens’ labels; Harvard Center for Morphometric Analysis, http://www.cma.mga.harvard.edu/fsl_atlas). To define those regions sensitive to the prospective valuation of information at the Scenario event, we took first-level SPM contrast images for the two subjective value modulators, and input these into a second-level factorial ANOVA with factors of Information and Effort. To define those regions sensitive to the value of information when it was definitively delivered, we took first-level SPM contrast images for the information value modulator at each of the Reveal and Outcome events, and input these into a second-level *t*-test for each event separately. In all analyses, we considered significant those voxels which survived cluster-wise corrections for family-wise error (FWE, *p* < .05), with a cluster-forming threshold of *p* = .001 (uncorrected). We additionally conducted the same analyses with the same contrasts at whole-brain level for an exploration of these effects without our a priori defined ROIs.

### Reporting summary

Further information on research design is available in the Nature Research Reporting Summary linked to this article.

## Supplementary information

Supplemental Material

Description of Additional Supplementary Files

Supplementary Data

Reporting Summary

## Data Availability

*t*-maps of the fMRI results reported above are available on NeuroVault (https://neurovault.org/collections/JOTABBXN/). Source data for the graphs and charts presented in this manuscript are available in the Supplementary Data.

## Code availability

Behavioural data were analysed with custom scripts in MATLAB R2015b (Mathworks Inc., US), R, and Stan. fMRI data were analysed with SPM12. The code used to generate the figures from this study are available on Zenodo (10.5281/zenodo.5598691).
