# Supplementary material for: Neurocomputational mechanisms underlying the subjective value of information
Source: Commun Biol. 2021 Dec 13;4:1346. doi: 10.1038/s42003-021-02850-3 (PMC8669024; doi:10.1038/s42003-021-02850-3)
Supplement: Supplementary file 1 — Supplemental Material [file 42003_2021_2850_MOESM1_ESM.pdf]

Neurocomputational mechanisms underlying the subjective value of information

Supplementary Material

Ariel X.-A. Goh [1,2], Daniel Bennett [3,4] , Stefan Bode [5], & Trevor T.-J. Chong [1,2,6,7*]

[1] Turner Institute for Brain and Mental Health, Monash University, Victoria 3800, Australia

[2] School of Psychological Sciences, Monash University, Victoria 3800, Australia

[3] Department of Psychiatry, Monash University, Victoria 3800, Australia

[4] Princeton Neuroscience Institute, Princeton University, Princeton NJ 08540, USA

[5] Melbourne School of Psychological Sciences, University of Melbourne, Victoria 3010, Australia

[6] Department of Neurology, Alfred Health, Melbourne, Victoria 3004, Australia

[7] Department of Clinical Neurosciences, St Vincent's Hospital, Melbourne, Victoria 3065, Australia

Supplementary Figures

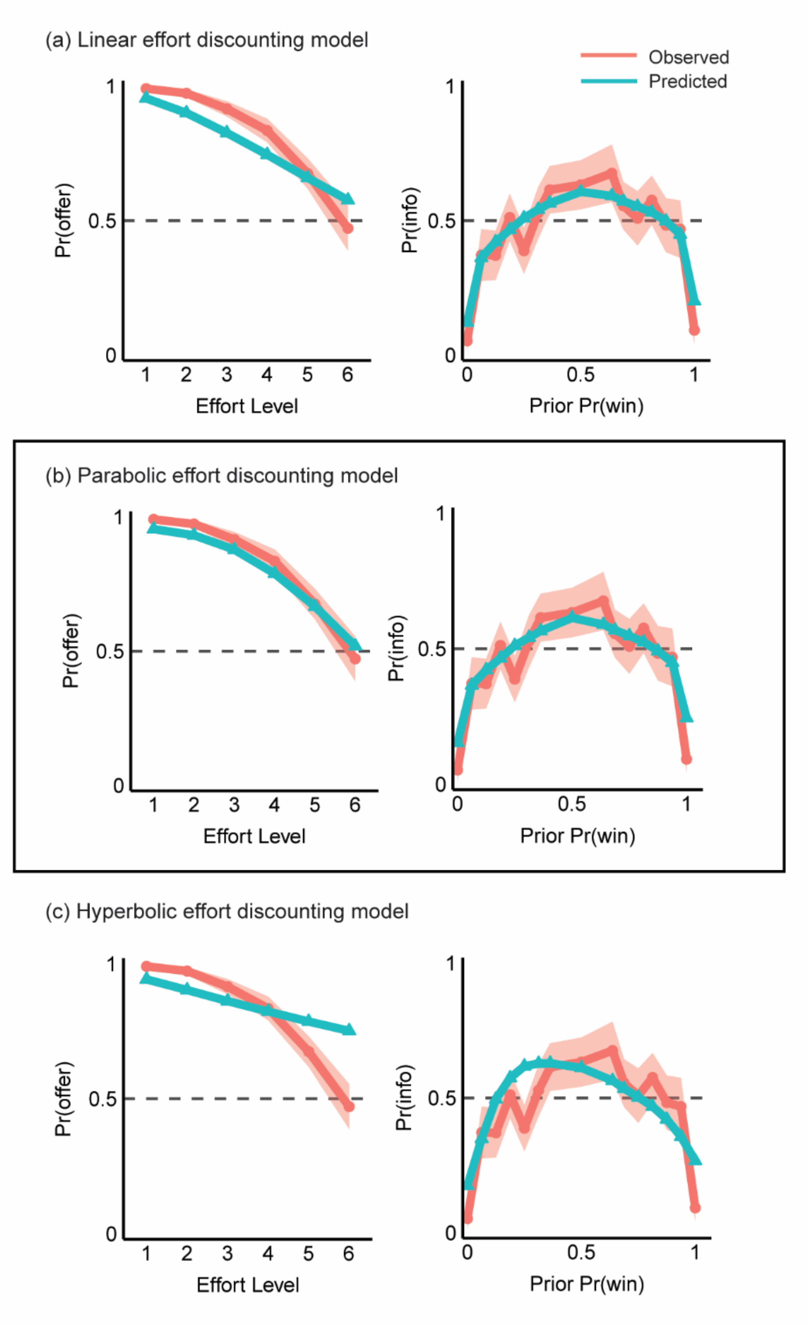

Supplementary Figure 1*. Posterior predictive checks for the (a) linear, (b) parabolic, and (c) hyperbolic effort discounting functions used to model choices in the effort discounting (left panels) and information seeking (right panels) tasks. Observed data are shown in orange, with shaded areas delineating 95% confidence intervals for the mean of the observed data in each condition. Predicted data are shown in blue. The parabolic family of effort discounting models provided the best fits, with a close correspondence between choice proportions predicted by the model and those observed in the data across both the effort discounting and information seeking tasks. Pr(offer) = Proportion of trials in which individuals chose the high reward offer in the effort discounting task. Pr(Info) = Proportion of trials in which individuals chose the informative option in the information-seeking task.*

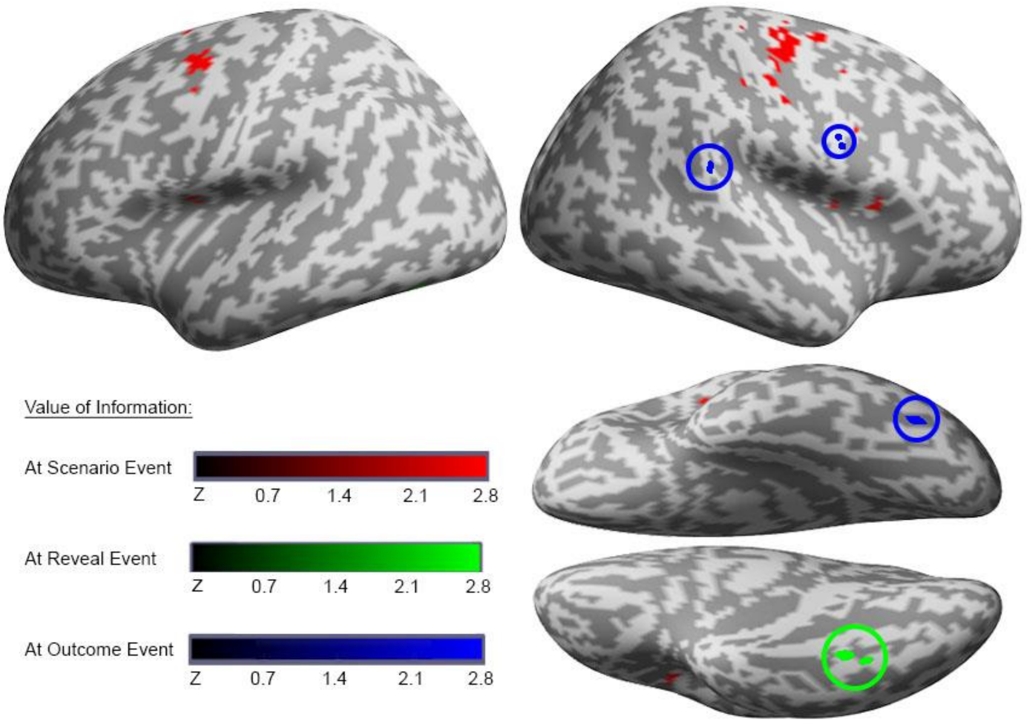

Supplementary Figure 2. *Results of an additional, exploratory whole-brain analysis on information value. Shown are clusters that were significant at a whole-brain level (corrected for family-wise error at a rate of p < .05) for the subjective value of information when prospectively evaluated (red); when delivered at the Reveal event (green); and when delivered at the Outcome event (blue).*

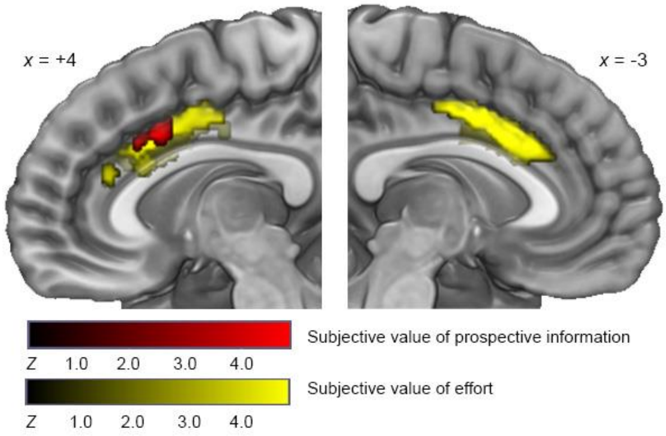

Supplementary Figure 3. *Activity within the right and left cingulate cortices encoded the subjective value of prospective information (red) and effort (yellow). Significant voxels indicate those that survived cluster-wise corrections for family-wise error (FWE, p < .05), with a cluster-forming threshold of p = .001 (uncorrected) in our ROI analysis.*

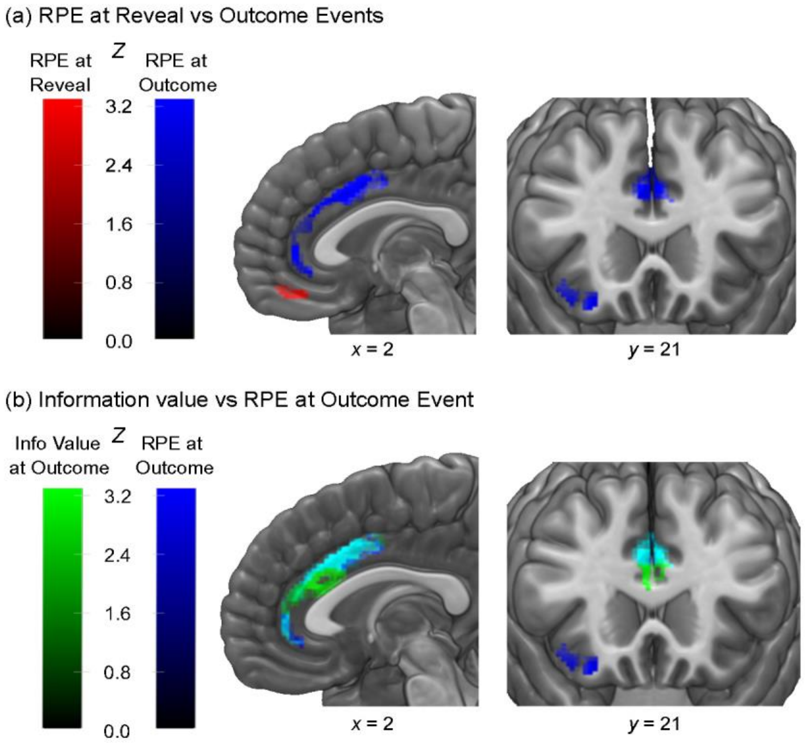

Supplementary Figure 4. *Seminal studies in the literature have addressed the similarity in activity evoked by information value and actual reward. Such studies have typically probed for reward-related activity in terms of reward prediction errors (RPEs)*[1-3]*, where typically* $RPE = Stake \cdot (P(win)_{post} - P(win)_{prior})$*. In our study,* $Stake = 10$ *credits on every trial;* $P(win)_{post}$ *denoted a win (1) or a loss (0); and* $P(win)_{prior}$ *was the prior probability of winning based on the Scenario event. We implemented a separate GLM that was similar to the main analysis for information value, but, instead of information value, we entered RPEs as a parametric modulator at the Reveal and Outcome events separately. Shown here are clusters of significant voxels that survived cluster-wise corrections for family-wise error (FWE, p < .05), with a cluster-forming threshold of p = .001 (uncorrected).*

*(a) RPEs evoked separate, non-overlapping clusters of activity at the Reveal and Outcome events (**Supplementary Table 5**). At the Reveal event, RPEs evoked activity within a single cluster within the ventromedial prefrontal cortex (vmPFC, red clusters). At the Outcome event (i.e., at the time of direct reward delivery), there was more extensive activity, extending across the anterior cingulate cortices, and right orbitofrontal cortex (blue clusters).*

The vmPFC, ACC and OFC in RPE have been shown in several studies to be sensitive to trial-by-trial increments in reward updating [4-9]*. Interestingly, however, these areas were engaged at different stages of our task, suggesting a differential sensitivity of these areas to the nature of the RPEs being encoded. Previous studies have shown that the vmPFC is sensitive to decision confidence* [10]*, which may explain its engagement during the Reveal event, which represented an intermediary stage in which the revealed cards signalled a potential for reward, but one that was yet to be realised. In contrast, RPEs only evoked ACC and OFC activity when rewards were definitively delivered. Obviously, the speculation that these areas are differentially sensitive to potential vs realised rewards remains to be examined in more targeted work.*

(b) At the Outcome event, there was an overlap in activity between areas that encoded information value (green) and RPE (blue; with overlap indicated in cyan). This would be expected for a region integrating information with reward outcomes. There were, however, voxels that were significantly engaged for information value that did not encode RPEs (within the anterior cingulate; green in Supp.Fig.4b), and those that encoded RPEs, but not information value (within the right orbitofrontal cortex; blue in Supp.Fig.4b). Overall, these results are in keeping with recent findings that information value shares similar neural substrates to reward, but that there may also be more specific mechanisms that encode the value of each entity.

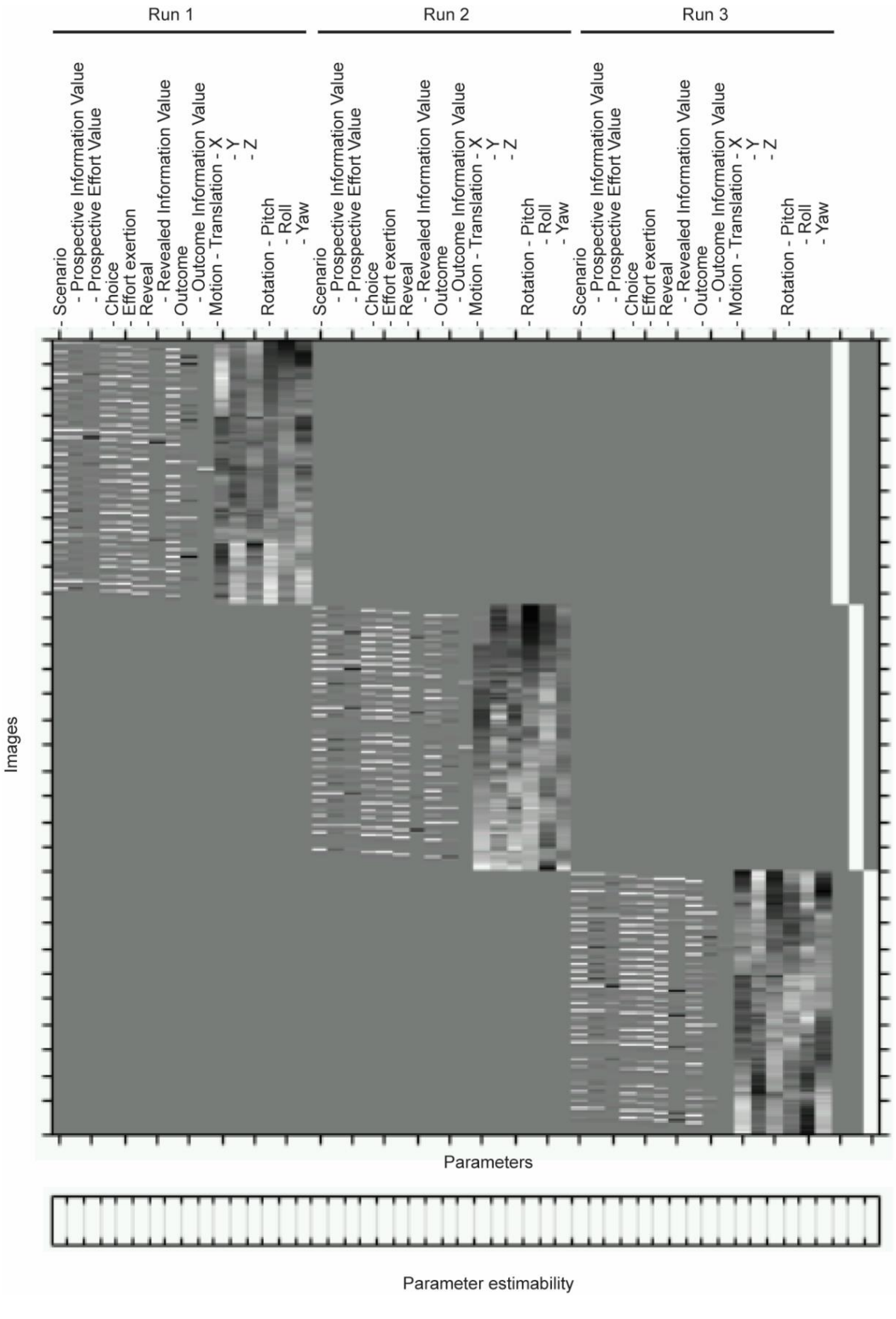

Supplementary Figure 5. *GLM design matrix for a representative participant.*

Supplementary Tables

Supplementary Table 1.

Pearson correlation matrix for parameters of the best-fitting computational model

	k_i	k_e	k_w	α	β
k_i	1	-	-	-	-
k_e	-.30	1	-	-	-
k_w	.60 *	- .19	1	-	-
α	.09	-.02	.05	1	-
β	-.45	.62 *	-.40	-.06	1

* denotes significant correlation, with $p < .05$ (Bonferroni-Holm-corrected for multiple comparisons)

Supplementary Table 2.

Results of model recovery analysis. Due to the large computational demands of running a model recovery analysis on the full set of 21 models, we performed a model recovery on a reduced set of four models. These included the best-fitting model, and three related parabolic effort discounting models, which factorially varied the type of entropy function (Shannon vs Rényi), and the effect of valence (present vs absent). Results confirmed that our model comparison procedure was able to correctly identify the true generative model on the basis of simulated data, with an accuracy in excess of 95% for each model. These results are based on 50 separate datasets per data-generating model.

		**Best-fitting model**			
		M 2.1	M 2.2	M 3.1	M 3.2 [§]
**Data-generating model** [†]	M 2.1	0.96	0.04	0	0
	M 2.2	0	1.0	0	0
	M 3.1	0	0	0.98	0.02
	M 3.2 [§]	0	0	0	1.0

[†] All models assume a parabolic effort discounting function. Model numbers correspond to Figure 3 in the main text.

- M 2.1: No valence modifier, Shannon entropy
- M 2.2: No valence modifier, Rényi entropy
- M 3.1: Valence modifier, Shannon entropy
- M 3.2: Valence modifier, Rényi entropy ([§] winning model)

Supplementary Table 3.

Results of parameter recovery analysis. We generated a synthetic dataset of the same size as the actual fMRI dataset, and assessed the rank-order correlation between the generative 'true' value of each parameter and the best-fitting value of that parameter by the same model. We quantified the recoverability of each parameter as the rank-order correlation between the generative parameter value and the fit parameter value. This statistic can be interpreted as a reliability coefficient for parameter estimation. All parameters were reliably recoverable ($p <$ .001 for all parameters).

**Parameter**	k_i	k_e	k_w	α	β
**Parameter estimation reliability**	0.84	0.94	0.68	0.62	0.94

Supplementary Table 4.

Variance inflation factors (VIF) for the events and regressors of interest in the general linear model used in the fMRI data analysis. Values are mean VIFs (with standard errors) across the group. Note that all VIFs are <4, indicating that multicollinearity between regressors was not an issue in our design.

	Scenario event	Info value regressor	Effort value regressor	Reveal event	Reveal regressor	Outcome event	Outcome regressor
**VIF**	2.06 (0.02)	1.04 (0.003)	1.04 (0.01)	3.05 (0.22)	2.00 (0.21)	2.31 (0.05)	1.63 (0.05)

Supplementary Table 5.

Areas activated by reward prediction errors (voxels survived cluster-wise corrections for family-wise error (p_{FWE} < .05), with an uncorrected cluster-forming threshold of p = .001).

**Cluster p_{FWE}**	***k***	***Z* value**	***x***	***y***	***z***	**Area**
**RPE at Reveal**						
0.010	73	3.77	-1	48	-17	Ventromedial prefrontal cortex
		3.41	2	40	-19	
**RPE at Outcome**						
< 0.001	494	4.66	8	38	18	Anterior cingulate cortex
		4.62	6	12	40	
		4.53	4	44	-1	
0.005	93	4.50	24	14	-19	Right orbitofrontal cortex
		4.00	28	26	-17	
		3.67	38	20	-13	

Supplementary Table 6.

Starting card configurations displayed at the Scenario event. For each positively-valenced configuration, we presented the corresponding negatively-valenced scenario. Of the 16 configurations, 7 were positively-valenced, 7 negatively-valenced, and 2 neutral.

**Number of Winning Cards**	**Number of Losing Cards**	$\mathbf{Pr}(\boldsymbol{win})$
6	2	1.0
4	1	0.94
4	2	0.88
3	1	0.81
4	3	0.75
3	2	0.69
1	0	0.64
3	3	0.5
0	0	0.5
0	1	0.36
2	3	0.31
3	4	0.25
1	3	0.19
2	4	0.13
1	4	0.06
2	6	0.0

Supplementary References

1 Bromberg-Martin, E. S. & Hikosaka, O. Midbrain dopamine neurons signal preference for advance information about upcoming rewards. *Neuron* **63**, 119-126 (2009).

2 Bromberg-Martin, E. S. & Hikosaka, O. Lateral habenula neurons signal errors in the prediction of reward information. *Nature Neuroscience* **14**, 1209-1216 (2011).

3 Brydevall, M., Bennett, D., Murawski, C. & Bode, S. The neural encoding of information prediction errors during non-instrumental information seeking. *Scientific Reports* **8**, 1-11 (2018).

4 Boorman, E. D., Behrens, T. E., Woolrich, M. W. & Rushworth, M. F. How green is the grass on the other side? Frontopolar cortex and the evidence in favor of alternative courses of action. *Neuron* **62**, 733-743 (2009).

5 Chib, V. S., Rangel, A., Shimojo, S. & O'Doherty, J. P. Evidence for a common representation of decision values for dissimilar goods in human ventromedial prefrontal cortex. *Journal of Neuroscience* **29**, 12315-12320 (2009).

6 Clithero, J. A. & Rangel, A. Informatic parcellation of the network involved in the computation of subjective value. *Social Cognitive and Affective Neuroscience* **9**, 1289-1132 (2014).

7 Hampton, A. N., Bossaerts, P. & O'Doherty, J. P. The role of the ventromedial prefrontal cortex in abstract state-based inference during decision making in humans. *Journal of Neuroscience* **26**, 8360-8367 (2006).

8 Kim, M. J., Gee, D. G., Loucks, R. A., Davis, F. C. & Whalen, P. J. Anxiety dissociates dorsal and ventral medial prefrontal cortex functional connectivity with the amygdala at rest. *Cerebral Cortex* **21**, 1667-1673 (2011).

9 McGuire, J. T. & Kable, J. W. Medial prefrontal cortical activity reflects dynamic re-evaluation during voluntary persistence. *Nature Neuroscience* **18**, 760-766 (2015).

10 Lebreton, M., Abitbol, R., Daunizeau, J. & Pessiglione, M. Automatic integration of confidence in the brain valuation signal. *Nature Neuroscience* **18**, 1159-1167 (2015).
