# Supplementary material for: Neurocomputational mechanisms underlying the subjective value of information
Source: Commun Biol. 2021 Dec 13;4:1346. doi: 10.1038/s42003-021-02850-3 (PMC8669024; doi:10.1038/s42003-021-02850-3)
Supplement: Supplementary file 2 — Description of Additional Supplementary Files [file 42003_2021_2850_MOESM2_ESM.pdf]

Description of Additional Supplementary Files

File name: Supplementary Data.
Description: Source data for the graphs and charts.
