# Supplementary material for: Neurocomputational mechanisms underlying the subjective value of information
Source: Commun Biol. 2021 Dec 13;4:1346. doi: 10.1038/s42003-021-02850-3 (PMC8669024; doi:10.1038/s42003-021-02850-3)
Supplement: Supplementary file 4 — Reporting Summary [file 42003_2021_2850_MOESM4_ESM.pdf]

nature research

Corresponding author(s): Trevor Chong

Last updated by author(s): Nov 18, 2021

Reporting Summary

Nature Research wishes to improve the reproducibility of the work that we publish. This form provides structure for consistency and transparency in reporting. For further information on Nature Research policies, see our Editorial Policies and the Editorial Policy Checklist.

Statistics

For all statistical analyses, confirm that the following items are present in the figure legend, table legend, main text, or Methods section.

n/a	Confirmed	
☐	☒	The exact sample size (*n*) for each experimental group/condition, given as a discrete number and unit of measurement
☐	☒	A statement on whether measurements were taken from distinct samples or whether the same sample was measured repeatedly
☐	☒	The statistical test(s) used AND whether they are one- or two-sided<br>*Only common tests should be described solely by name; describe more complex techniques in the Methods section.*
☐	☒	A description of all covariates tested
☐	☒	A description of any assumptions or corrections, such as tests of normality and adjustment for multiple comparisons
☐	☒	A full description of the statistical parameters including central tendency (e.g. means) or other basic estimates (e.g. regression coefficient) AND variation (e.g. standard deviation) or associated estimates of uncertainty (e.g. confidence intervals)
☐	☒	For null hypothesis testing, the test statistic (e.g. F, t, r) with confidence intervals, effect sizes, degrees of freedom and P value noted<br>*Give P values as exact values whenever suitable.*
☒	☐	For Bayesian analysis, information on the choice of priors and Markov chain Monte Carlo settings
☐	☒	For hierarchical and complex designs, identification of the appropriate level for tests and full reporting of outcomes
☐	☒	Estimates of effect sizes (e.g. Cohen's d, Pearson's r), indicating how they were calculated

Our web collection on statistics for biologists contains articles on many of the points above.

Software and code

Policy information about availability of computer code

Data collection	Data were collected with custom scripts implemented in the Psychophysics Toolbox implemented in MATLAB R2015b (Mathworks Inc., US).
Data analysis	Behavioural data were analysed with custom scripts in MATLAB R2015b (Mathworks Inc., US), R, and Stan. fMRI data were analysed with SPM12.

For manuscripts utilizing custom algorithms or software that are central to the research but not yet described in published literature, software must be made available to editors and reviewers. We strongly encourage code deposition in a community repository (e.g. GitHub). See the Nature Research guidelines for submitting code & software for further information.

Data

Policy information about availability of data

All manuscripts must include a data availability statement. This statement should provide the following information, where applicable:

- Accession codes, unique identifiers, or web links for publicly available datasets
- A list of figures that have associated raw data
- A description of any restrictions on data availability

t-maps of the fMRI results reported above are available on NeuroVault (https://neurovault.org/collections/JOTABBXN/). Source data for the graphs and charts presented in this manuscript are available in the Supplementary Data.

Field-specific reporting

Please select the one below that is the best fit for your research. If you are not sure, read the appropriate sections before making your selection.

☐ Life sciences ☒ Behavioural & social sciences ☐ Ecological, evolutionary & environmental sciences

For a reference copy of the document with all sections, see nature.com/documents/nr-reporting-summary-flat.pdf

Behavioural & social sciences study design

All studies must disclose on these points even when the disclosure is negative.

Study description	Quantitative, experimental, model-based fMRI study
Research sample	Healthy young adults with approximately balanced gender representation (12 male, 14 female).
Sampling strategy	Volunteers responded to an advertisement for the study. Sample size was comparable to other fMRI studies done in this area.
Data collection	Participants completed an effort-based decision-making task outside the scanner, and an in-scanner information-seeking task. Order was counterbalanced across participants. A researcher was present to familiarise participants to the task.
Timing	January to May 2018
Data exclusions	4 of 30 participants were excluded for failing to comprehend or comply with task instructions.
Non-participation	None.
Randomization	Not applicable.

Reporting for specific materials, systems and methods

We require information from authors about some types of materials, experimental systems and methods used in many studies. Here, indicate whether each material, system or method listed is relevant to your study. If you are not sure if a list item applies to your research, read the appropriate section before selecting a response.

Materials & experimental systems

n/a	Involved in the study	
☒	☐	Antibodies
☒	☐	Eukaryotic cell lines
☒	☐	Palaeontology and archaeology
☒	☐	Animals and other organisms
☐	☒	Human research participants
☒	☐	Clinical data
☒	☐	Dual use research of concern

Methods

n/a	Involved in the study	
☒	☐	ChIP-seq
☒	☐	Flow cytometry
☐	☒	MRI-based neuroimaging

Human research participants

Policy information about studies involving human research participants

Population characteristics	See above
Recruitment	Volunteers responded to an advertisement for the study.
Ethics oversight	Monash University Human Research Ethics Committee

Note that full information on the approval of the study protocol must also be provided in the manuscript.

Magnetic resonance imaging

Experimental design

Design type	Model-based fMRI
Design specifications	3 runs of 32 trials per subject. Each run lasted 16:49

Behavioral performance measures

Binary choice measures

Acquisition

Imaging type(s)

Functional MRI

Field strength

3T

Sequence & imaging parameters

Stimuli were displayed on an MRI-compatible monitor positioned at the head of the scanner bore, and participants viewed the monitor through a mirror mounted on a 32-channel head coil. Functional data were acquired with a T2*-weighted gradient echo-planar imaging (EPI) sequence using interleaved slice acquisition (TR 2,200 ms; TE 30 ms; flip angle 90°; 38 contiguous slices with a slice thickness of 3.0 mm without an interslice gap; voxel size 3.0 mm3 on a base matrix of 64 64 pixels, oriented along the AC-PC line).

Area of acquisition

Whole brain

Diffusion MRI ☐ Used ☒ Not used

Preprocessing

Preprocessing software

FMRIPREP standard pre-processing pipeline - see paper for details

Normalization

Spatial normalisation to the ICBM 152 Nonlinear Asymmetrical template version 2009c was performed through nonlinear registration with the antsRegistration tool of ANTs v2.1.0, using brain-extracted versions of both T1w volume and template. Brain tissue segmentation of cerebrospinal fluid (CSF), white-matter (WM) and gray-matter (GM) was performed on the brain-extracted T1w using fast (FSL v5.0.9).

Normalization template

ICBM 152 Nonlinear Asymmetrical template version 2009c

Noise and artifact removal

Physiological noise regressors were extracted applying CompCor. Principal components were estimated for the two CompCor variants: temporal (tCompCor) and anatomical (aCompCor). A mask to exclude signal with cortical origin was obtained by eroding the brain mask, ensuring it only contained subcortical structures. Six tCompCor components were then calculated including only the top 5% variable voxels within that subcortical mask. For aCompCor, six components were calculated within the intersection of the subcortical mask and the union of CSF and WM masks calculated in T1w space, after their projection to the native space of each functional run. Frame-wise displacement was calculated for each functional run using the implementation of Nipype.

Volume censoring

Functional data was slice-time corrected using 3dTshift from AFNI v16.2.07 and motion corrected using mcflirt (FSL v5.0.9). This was followed by co-registration to the corresponding T1w using boundary-based registration with six degrees of freedom, using bbregister (FreeSurfer v6.0.1). Motion correcting transformations, BOLD-to-T1w transformation and T1w-to-template (MNI) warp were concatenated and applied in a single step using antsApplyTransforms (ANTs v2.1.0) using Lanczos interpolation.

Statistical modeling & inference

Model type and settings

Data were analysed using SPM12 (Wellcome Department of Imaging Neuroscience, Institute of Neurology, London, United Kingdom; http://www.fil.ion.ucl.ac.uk/spm), implemented in MATLAB. Each participant’s data were modelled using fixed-effects analyses. The effects of the experimental paradigm were estimated for each participant on a voxel-by-voxel basis using the principles of the general linear model (GLM). Predictor functions were formed by modelling the onsets of the events of interest with a stick (delta) function convolved with the canonical haemodynamic response function. Low-frequency noise was removed with a 128 s high-pass filter. The GLM included three regressors of interest: the Scenario event, the Reveal event, and the Outcome event, each of which was associated with a parametric modulator (see below). Other regressors which were included, but not analysed, included the motor events (i.e., the Choice and Effort events), and the onsets of the catch trials and their outcomes. The six head motion parameters derived during realignment (three translations and three rotations) were incorporated as additional nuisance regressors.

The main focus of this model-based fMRI study was to determine the neurocomputational mechanisms underlying: (1) the subjective valuation of information, and (2) the reduction of uncertainty across individual participants. To address the first goal, we computed the subjective value of information (i.e., k_i·I + k_w·W) for the chosen option on every trial for every participant using the parameters from our best-fitting model. In addition, we computed the subjective value of effort (i.e., k_e·E) for every trial using the same model. We then entered these two SVs as orthogonalised, parametric modulators for the Scenario event-related regressor.

To address the second goal, we computed the subjective value of information when it was finally delivered at the Reveal or Outcome screens. As for the first goal, the subjective value of information was defined through the winning model as k_i·I + k_w·W, which represents the amount by which uncertainty was reduced (as defined by the Rényi entropy function with a participant-specific alpha parameter), added to the valence of information. These subjective values were then entered as parametric modulators for the Reveal and Outcome events separately. Regression coefficients were estimated at the subject level using the standard restricted minimum-likelihood estimation implemented in SPM12. Variance inflation factors for all of our regressors were < 4, indicating that multicollinearity between regressors was not an issue in our design. A GLM design matrix for a representative participant is provided in Supplementary Figure 5.

Effect(s) tested

SPM contrast images from the first level were then taken to a second-level group analysis. To define those regions sensitive to the prospective valuation of information at the Scenario event, we took first-level SPM contrast images for the two subjective value modulators, and input these into a second-level factorial ANOVA with factors of Information and Effort. To define those regions sensitive to the value of information when it was definitively delivered, we took first-level SPM contrast images for the information value modulator at each of the Reveal and Outcome events, and input these into a second-level t-test for each event separately.

Specify type of analysis: ☐ Whole brain ☐ ROI-based ☒ Both

Anatomical location(s)

We restricted our analyses to all voxels within regions-of-interest (ROIs) comprising the ACC, vmPFC, OFC and VS using the Harvard-Oxford Cortical and Subcortical Structural Atlas (corresponding to the 'anterior cingulate', 'medial frontal', 'frontal orbital' and 'nucleus accumbens' labels; Harvard Center for Morphometric Analysis, http://www.cma.mga.harvard.edu/fsl_atlas).

Statistic type for inference
(See Eklund et al. 2016)

In all analyses, we considered significant those voxels which survived cluster-wise corrections for family-wise error (FWE, p < .05), with a cluster-forming threshold of p = .001 (uncorrected). We additionally conducted the same analyses with the same contrasts at whole-brain level for an exploration of these effects without our a priori defined ROIs.

Correction

FWE

Models & analysis

n/a	Involved in the study
☒	☐ Functional and/or effective connectivity
☒	☐ Graph analysis
☒	☐ Multivariate modeling or predictive analysis
